# Cumulative exposure to cannabis and hippocampus MRI in middle age: results from the coronary artery risk development in young adults (CARDIA) study

**DOI:** 10.1038/s41398-026-04096-1

**Published:** 2026-05-19

**Authors:** Barbara Schilling, Baptiste Pasquier, Martine Elbejjani, Jared Reis, Jamal S. Rana, Kali Tal, Lenore J. Launer, Stéphanie Baggio, Stephen Sidney, Nick Bryan, Kristine Yaffe, Reto Auer, Julian Jakob

**Affiliations:** 1 Institute of Primary Health Care (BIHAM), University of Bern, Bern, Switzerland; 2 American University of Beirut, Beirut, Lebanon; 3 National Heart, Lung, and Blood Institute, Bethesda, MD USA; 4 Kaiser Permanente Northern California, Department of Cardiology and Division of Research, Oakland, CA USA; 5 National Institute on Aging, Bethesda, MD USA; 6 Institute of Psychology, University of Lausanne, Lausanne, Switzerland; 7 Kaiser Permanente Division of Research, Oakland, CA USA; 8 University of Pennsylvania, Philadelphia, PA USA; 9 University of California San Francisco, San Francisco, CA USA; 10 Center for Primary Care and Public Health (Unisanté), University of Lausanne, Lausanne, Switzerland; 11 Department of Paediatrics, University Hospital Bern, Inselspital, Bern, Switzerland

**Keywords:** Diagnostic markers, Addiction

## Abstract

Cannabis was previously associated with worse memory function in men but not in women. As the hippocampus is crucial in the formation and retrieval of memory, we studied if cumulative exposure to cannabis is associated with differences in the hippocampal tissue volume, fractional anisotropy (FA) and cerebral brain perfusion (CBF) by MRI, overall and by sex, stratified by ever tobacco smoking, in multivariable adjusted linear regression models in both sexes. We included participants of the CARDIA cohort, followed since 1985, with cannabis assessed during each follow up. Categories of self-reported cumulative exposure were never, <0.5, 0.5–<2, and >2 cannabis-years, where 1 cannabis-year=365 days of use. We included 648 participants: 52% were women; mean age was 55 years, 86% reported ever using cannabis and 48% ever smoking tobacco. There was no difference in mean hippocampal volume according to greater cumulative use of cannabis. The coefficient of hippocampal volume in participants never smoking tobacco reporting >2 cannabis-years was −37.99mm^3^ (95% CI −201.08–125.09) compared to never users. There was no significant difference when stratifying by sex or ever tobacco exposure, or for FA or CBF. Cumulative cannabis exposure over 30 years was not associated with hippocampal volume, integrity or blood flow in middle age. The differences in memory function in cannabis users are likely not attributable to the hippocampus only. Future studies should assess further neuronal mechanisms and social determinants associated with cognition in cannabis users.

## Introduction

Cannabis is the most widely used illicit drug and its use is increasing in midlife and older adults. An estimated 219 million people worldwide used cannabis in 2021, representing 4% of the global adult population, and the number of people who use cannabis has increased by 21% over the past decade [1]. Some evidence suggests cannabis use may impair cognition as well as brain structure [2, 3]. These findings are important because cognitive impairment and alterations in brain structure (such as reduced hippocampal volume) are risk factors for neurodegenerative disorders, causing a significant burden on health care [4, 5].

In a previous investigation, we found self-reported cumulative cannabis use over lifetime was associated with worse verbal memory in men [6]. We know that the hippocampus plays a pivotal role in the formation and retrieval of memory, so cannabis use might affect hippocampus structure and function [7, 8]. Previous literature on the topic of cannabis and hippocampus structure and function found smaller hippocampi in cannabis users compared to non-users [9–15], some reported white matter alterations around the hippocampus as measured by diffusion tensor imaging studies (DTI) [16–18] [18–20], and one study reported changes in regional cerebral blood flow (CBF) in (para)hippocampal regions in frequent cannabis users [21], while other studies found no association with cannabis use [22–24]. These studies reported on other brain regions (e.g. amygdala, prefrontal cortex) as well, and some found an association with cumulative exposure to cannabis, but many did not find an association [9–11, 25–30]. Unfortunately, most previous studies assessed cannabis exposure retrospectively at only one time point only, included only few participants or failed to adjust for relevant covariables associated with both cannabis use and brain health outcomes (e.g. education, other substance use, tobacco smoking).

As many previous studies suffer from methodological limitations, evidence on the associations between cumulative cannabis use and hippocampus outcomes is limited, with some studies reporting associations, while other studies report none. We need larger studies with a longitudinal design to strengthen the evidence on the associations between cumulative cannabis use and hippocampus structure and function in the general population. We thus aim at investigating alterations in hippocampal volume by MRI, connectivity in the fornix using fractional anisotropy (FA), and change in cerebral blood flow in the hippocampus (CBF) in a large cohort of Black and White women and men followed for 30 years with repeated assessments of cannabis use to compute cumulative cannabis exposure and data on many relevant covariables, such as education and other substance use including tobacco smoking.

## Methods

### Study design and sample

We used data from the Coronary Artery Risk Development in Young Adults Study, a population-based cohort of 5115 adults aged 18–30 years at baseline and followed up for 35 years. At the year 30 exam (2015–2016), 662 participated in the brain MRI sub-study. A detailed description of the cohort has been published elsewhere [31]. We excluded participants with a history of stroke or Transient Ischemic Attack (TIA) from the main analysis based on previous studies showing both changes in brain structure as well as white matter integrity in patients after TIA or stroke [32–35]. All participants gave written informed consent before entering the study and at each visit, and the institutional review boards at each site granted approval for the study [6].

### Cannabis exposure: current and cumulative

Current cannabis use was assessed at each in-person CARDIA visit (at baseline and during their 2, 5, 7, 10, 15, 20, 25, and 30 year follow-up visits) with the following survey questions: “During the last 30 days, on how many days did you use cannabis?” Direct self-reported cumulative exposure was assessed with the question: “About how many times in your lifetime have you used cannabis?” Current and lifetime use were used to compute cannabis-years: one year of exposure was equivalent to 365 days of cannabis use. Current use at each visit (the number of days of cannabis was used in the month before each visit) was assumed to indicate the average number of days of use in the months before and after each visit. The total number of days the participant used cannabis over follow-up was summed to estimate self-reported cumulative cannabis use. E.g. if a participant reported 30 days of cannabis use in the past 30 days (daily use), we computed one cannabis-year per year between visits (5 years between visits thus leads to 5 cannabis-years for this period). Similarly, if a participant reported 6 days of use in 30 days (use every five days), we computed 0.2 cannabis-years per year between visits (1 cannabis-year in 5 years, see Online Appendix for detailed information). Whenever direct self-reported lifetime use was higher than the computed estimates, the estimate was adjusted upwards. At the Year 30 visit, acute cannabis use was assessed with the following survey question: “Did you use cannabis in the last 24 h?” [36] As previously, participants were classed into existing categories of self-reported cumulative [36]: never use; 1 day to <0.5 cannabis-years; 0.5 to <2.0 cannabis-years and 2.0 or more cannabis-years [6].

### Brain MRI data

The Brain MRI sub-study at Year 30 had the aim of achieving a balanced sample within sex-race groups from three of the CARDIA field centers: Birmingham, AL, Minneapolis, MN, and Oakland, CA. Exclusion criteria were a contra-indication to MRI or a body size that was too large for the MRI tube bore [37]. Multi-modal MRI scans including structural and diffusion weighted imaging were obtained using 3-Tesla MR scanners: Siemens 3 T Tim Trio/VB15 platform in Minneapolis, MN and Oakland, CA and Philips 3 T Achieva/2.6.3.6 platform in Birmingham, AL and sent to the MRI reading center at the University of Pennsylvania (Section of Biomedical Image Analysis, Department of Radiology) [38]. Scanner performance was monitored with quarterly Alzheimer’s Disease Neuroimaging Initiative phantom acquisitions, which showed stability of phantom measurements throughout the study.

Detailed protocols have been published. Briefly: the CARDIA brain MRI imaging protocol included sagittal 3D T1 (TR = 1900 ms, TE = 2.89 ms, FOV = 250 mm, thickness = 1 mm, slices = 176, native resolution = 1 mm isotropic), T2 ((TR = 3200 ms, TE = 409 ms, FOV = 250 mm, thickness = 1 mm, slices = 176, native resolution = 1 mm isotropic), FLAIR (TR = 6000 ms, TE = 285 ms, FOV = 258 mm, thickness = 1 mm, slices = 160, native resolution = 1 mm isotropic) and 30-directional DTI (TR = 7400 ms, TE = 82 ms, FOV = 246 mm, thickness = 2.2 mm, slices = 64, native resolution = 2.2 mm isotropic) [38].

#### Volumetry

Structural MRI scans were preprocessed in several steps including histogram normalization, bias field correction of raw data, template registration, and semiautomated tissue segmentation based on the Multi-atlas region Segmentation utilizing Ensembles (MUSE) framework [39] into grey matter (GM), white matter (WM), and cerebrospinal fluid (CSF). Intra-cranial volume (ICV), and regions of interest (ROIs) were estimated using DeepMRSeg (https://github.com/CBICA/DeepMRSeg) [40], a convolutional neural network trained for accurate segmentation from T1-weighted scans. We used ROI 47 and 48 for the hippocampus, 89 and 90 for the fornix, 90, 91, 136, 137, 142, 143, 146, 147 for the prefrontal cortex, and 31 and 32 for the amygdala. We used ICV to normalize for head size [38, 41].

#### Fractional anisotropy

Voxel-wise diffusion maps including fractional anisotropy (FA) were derived from raw Diffusion Tensor Imaging (DTI) images using standard methods in three steps: 1) Local-PCA denoising, implemented in Diffusion Imaging in Python (DIPY) [42]; 2) motion and distortion correction using FSL’s “eddy” tool (http://www.fmrib.ox.ac.uk/fsl) [43]; and 3) bias field correction with N4BiasCorrection [44] in the Advanced Normalization Tools (ANTs) software package (http://stnava.github.io/ANTs/) [45]. Each subject’s T1 was then registered to their DTI space and the transformations were concatenated to map ROI labels directly to DTI space [39]. DTI measures were extracted from the whole WM. The FA measure estimates the degree or uniformity to which water diffuses along the direction of myelinated tracks in the white matter. The FA scores range from 0–1 with lower scores indicating worse white matter integrity. Because FA measures the integrity of white matter, we defined the fornix as hippocampal outcome for this analysis, the fornix being the major output pathway of the hippocampus playing a crucial role in its communication with other brain areas, particularly those involved in memory and emotion processing [46].

#### Cerebral brain perfusion (CBF)

Volume of flow per unit brain mass per unit time (mL/100 g/min) was measured with an axial pseudo-Continuous Arterial Spin Labeling (pCASL) [38].

### Covariables

Demographic variables included age, sex, race, study center and level of education. Level of education was defined as the highest educational grade attained by Year 30.

Cigarette smoking was evaluated during each in-person visit and through yearly telephone surveys. These data were used to estimate cumulative lifetime exposure to tobacco smoking in terms of pack-years. One pack-year of exposure corresponding to smoking one pack of cigarettes per day for one year [47, 48]. We estimated lifetime alcohol consumption in drink-years. Defining one drink-year as the amount of alcohol consumed in one year by a person consuming one drink per day [49]. Men who reported 5 or more drinks on a single occasion and women who reported 4 or more were categorized as having acute heavy exposure to alcohol (bingeing). According to this information, we estimated cumulative lifetime bingeing episodes. We estimated total number of lifetime exposure to cocaine (including other forms of cocaine, such as crack, powder, or freebase), amphetamines (speed, uppers, or methamphetamines), and heroin based on repeated assessments [50, 51]. Acute intoxication with any substance was not queried prior to the MRI. Self-reported depression was measured every 5 years starting at the year 5 visit using the Center for Epidemiologic Studies Depression scale, we used a score of >= 16 as cut-off for a binary variable [52]. We used the body mass index (BMI) as a measure for overall health and cardiovascular risk factor. Variables were assessed for normality and transformed where necessary. More detailed information about the assessment of covariables is outlined in the supplementary material.

### Statistical analysis

We used descriptive statistics to assess participant characteristics with different levels of exposure to cannabis at the year 30 visit. To examine the association between cannabis use, and hippocampal structure and connectivity, we set up a series of linear regression models. We present results in the overall sample, and then stratifying by ever tobacco smoking due to multicollinearity with cannabis exposure and based on the known harmful effects of tobacco smoking on the brain [41]. We tested three adjustment models in sequence. The first models were unadjusted (besides accounting for total intracranial volume in hippocampal volume analyses). The second models controlled for the covariables used to achieve a balance of sampling in CARDIA: age, sex, ethnicity, years of education and study center. The third models additionally controlled for covariables potentially associated with cannabis use, cognition and brain volume, based on clinical plausibility: alcohol use (current, cumulative and binge drinking), current cocaine, amphetamine use, and heroin, body mass index (BMI) and depression. The models for hippocampus volume, FA and CBF were pre-specified in a CARDIA study proposal. We present p-values for trend across categories, testing joint hypotheses on multiple exposure categories.

Exploratory secondary outcomes included measures of volume, FA and CBF for the prefrontal cortex and the amygdala in the fully adjusted model. These analyses should be considered hypothesis-generating, and not confirmatory.

### Sensitivity analyses

We performed the following sensitivity analyses to strengthen the confidence in main models:

1. Testing the left and right hippocampus separately, as previous studies found differences between hemispheres [9, 13, 14, 25].
2. Adjusting for the mirror star tracing test at Year 2 to address potential reverse causation. At Year 2, the mirror star tracing test elicited a reactive blood pressure response. Although initially intended as a stressor to measure blood pressure, other studies have suggested it can be used as a measure of executive function [31, 53, 54]. We used it to adjust for baseline cognitive function.
3. Adjusting for extended cardiovascular risk factor measurements (blood pressure, blood cholesterol levels, fasting glucose level, diabetes mellitus).
4. Not excluding participants with a history of stroke or TIA for main analyses.
5. Including only participants with ongoing current (in past 30 days) cannabis use or without current cannabis use at their year 30 visit.
6. Cumulative cannabis use modeled flexibly as non-linear term using restricted cubic splines.

## Results

Of the 3357 (66% of initial sample of 5115) participants assessed at the Year 30 visit, 662 (20% of the population at Year 30) underwent the MRI sub-study. After exclusion of participants with prior stroke or TIA (N = 14), 648 remained in our sample; 340 reported never smoking (52% women, mean age 55 years, 77.9% reported ever cannabis use) and 308 reported ever smoking tobacco (40% women, mean age 55 years, 94% reported ever cannabis use). Participants across the cumulative cannabis categories varied in many characteristics (Table 1a and b, stratified by ever tobacco smoking; Appendix Table 1 for combined dataset).

**Table 1:** (a): Characteristics of 340 CARDIA participants that never reported smoking tobacco with participation at the Year 30 MRI sub-study excluding participants with a history of stroke or TIA. (b): Characteristics of 308 CARDIA participants that ever reported smoking tobacco with participation at the Year 30 MRI sub-study excluding participants with a history of stroke or TIA.

Never Tobacco Smoking:Variable	All	Never	Ever Cannabis Use^a^			p-value^b^
			1 day to 0.5 cannabis years	0.5 to < 2 cannabis years	> 2 cannabis years	
N (%)	340 (100.0%)	75 (22.1%)	175 (51.5%)	59 (17.4%)	31 (9.1%)	
(**a**)						
**Demographics**						
Age years, mean (SD)	55 (3)	55 (4)	55 (4)	56 (4)	56 (4)	0.14
Race, N (Col. %)^c^						**0.032**
Black women	67 (19.7%)	20 (26.7%)	37 (21.1%)	4 (6.8%)	6 (19.4%)	
Black men	48 (14.1%)	12 (16.0%)	21 (12.0%)	9 (15.3%)	6 (19.4%)	
White women	110 (32.4%)	23 (30.7%)	63 (36.0%)	20 (33.9%)	4 (12.9%)	
White men	115 (33.8%)	20 (26.7%)	54 (30.9%)	26 (44.1%)	15 (48.4%)	
Education years, mean (SD)	16 (2)	16 (3)	17 (2)	16 (2)	16 (2)	0.24
Study center, N (Col. %)						**<0.001**
Birmigham, AL	97 (28.5%)	37 (49.3%)	43 (24.6%)	14 (23.7%)	3 (9.7%)	
Minneapolis, MI	109 (32.1%)	27 (36.0%)	53 (30.3%)	24 (40.7%)	5 (16.1%)	
Oakland, CA	134 (39.4%)	11 (14.7%)	79 (45.1%)	21 (35.6%)	23 (74.2%)	
**Substance use exposure**						
Tobacco smoking, N (Col. %)						n/a
never smoker	340 (100.0%)	75 (100.0%)	175 (100.0%)	59 (100.0%)	31 (100.0%)	
past smoker	0 (0.0%)	0 (0.0%)	0 (0.0%)	0 (0.0%)	0 (0.0%)	
current smoker	0 (0.0%)	0 (0.0%)	0 (0.0%)	0 (0.0%)	0 (0.0%)	
Cumulative tobacco exposure among ever smokers/ tobacco pack-years^e^	0	0	0	0	0	n/a
Alcohol use	13.4 (20.4)	4.3 (9.3)	11.7 (14.5)	17.5 (17.0)	36.7 (43.5)	**<0.001**
Cumulative alcohol use among ever drinkers/ drink-years^f^, mean (SD)						
Cum. lifetime binge episodes^g^/ binge drinking days, mean (SD)	160 (464)	72 (350)	103 (236)	158 (278)	708 (1143)	**<0.001**
Cannabis use status, N (Col. %)^d^						**<0.001**
never user	75 (22.1%)	75 (100.0%)	0 (0.0%)	0 (0.0%)	0 (0.0%)	
past user	231 (67.9%)	0 (0.0%)	174 (99.4%)	47 (79.7%)	10 (32.3%)	
current user	34 (10.0%)	0 (0.0%)	1 (0.6%)	12 (20.3%)	21 (67.7%)	
Cumulative cannabis exposure / cannabis years^a^, mean (SD)	1.2 (3.6)	0.0 (0.0)	0.07 (0.08)	1.2 (0.5)	9.9 (7.9)	**<0.001**
Illicit drug use						**<0.001**
Current use^h^ −Cocaine, crack, speed or methamphetamine, N (Col. %)	7 (2.1%)	0 (0.0%)	1 (0.6%)	0 (0.0%)	6 (19.4%)	
−Heroin, N (Col. %)	0 (0.0%)	0 (0.0%)	0 (0.0%)	0 (0.0%)	0 (0.0%)	
**Anthropomorphic variable** BMI^i^, mean (SD)	28.0 (4.6)	28.8 (4.5)	27.5 (4.7)	28.6 (4.7)	27.9 (4.6)	0.13
**Psychological variable** Depression, current CES-D > = 16/30^j^, N (Col. %)	161 (12.6%)	34 (11.3%)	82 (11.8%)	26 (14.7%)	19 (18.3%)	0.48

Ever Tobacco Smoking: Variable	All	Never	Ever Cannabis Use^a^			p-value^b^
			1 day to 0.5 cannabis years	0.5 to < 2 cannabis years	> 2 cannabis years	
N (%)	308 (100.0%)	18 (5.8%)	105 (34.1%)	98 (31.8%)	87 (28.2%)	
(**b**)						
**Demographics**						
Age years, mean (SD)	55 (3)	54 (4)	55 (3)	56 (3)	55 (4)	0.06
Race, N (Col. %)^c^						**<0.001**
Black women	72 (23.4%)	10 (55.6%)	26 (24.8%)	22 (22.4%)	14 (16.1%)	
Black men	69 (22.4%)	2 (11.1%)	19 (18.1%)	12 (12.2%)	36 (41.4%)	
White women	3 (16.7%)	42 (40.0%)	34 (34.7%)	12 (13.8%)	91 (29.5%)	
White men	76 (24.7%)	3 (16.7%)	18 (17.1%)	30 (30.6%)	25 (28.7%)	
Education years, mean (SD)	15 (3)	15 (3)	156 (3)	15 (2)	14 (2)	**<0.001**
Study center, N (Col. %)						0.002
Birmigham, AL	75 (24.4%)	12 (66.7%)	27 (25.7%)	19 (19.4%)	17 (19.5%)	
Minneapolis, MI	141 (45.8%)	5 (27.8%)	46 (43.8%)	48 (49.0%)	42 (48.3%)	
Oakland, CA	92 (29.9%)	1 (5.6%)	32 (30.5%)	31 (31.6%)	28 (32.2%)	
**Substance use exposure**						
Tobacco smoking, N (Col. %)						**<0.001**
never smoker						
past smoker	226 (73.4%)	14 (77.8%)	82 (78.1%)	81 (82.7%)	49 (56.3%)	
current smoker	82 (26.6%)	4 (22.2%)	23 (21.9%)	17 (17.3%)	38 (43.7%)	
Cumulative tobacco exposure among ever smokers/ tobacco pack-years^e^, mean (SD)	11.0 (12.5)	7.9 (13.5)	9.7 (12.64)	10.3 (11.1)	13.9 (13.2)	0.002
Alcohol use						0.50
Cumulative alcohol use among ever drinkers/ drink-years^f^, mean (SD)	30.6 (40.1)	11.3 (16.9)	18.7 (25.8)	29.0 (32.2)	50.80 (55.2)	
Cum. lifetime binge episodes^g^/ binge drinking days, mean (SD)	509 (969)	126 (255)	195 (443)	456 (721)	1029 (1’444)	**<0.001**
Cannabis use status, N (Col. %)^d^						**<0.001**
never user	18 (5.8%)	18 (100.0%)	0 (0.0%)	0 (0.0%)	0 (0.0%)	
past user	220 (71.4%)	0 (0.0%)	104 (99.0%)	81 (82.7%)	35 (40.2%)	
current user	70 (22.7%)	0 (0.0%)	1 (1.0%)	17 (17.3%)	52 (59.8%)	
Cumulative cannabis exposure / cannabis years^a^, mean (SD)	2.5 (4.0)	0.0 (0.0)	0.1 (0.1)	1.3 (0.5)	7.3 (4.9)	**<0.001**
Illicit drug use						0.071
Current use^h^ −Cocaine, crack, speed or methamphetamine, N (Col. %)	15 (4.9%)	1 (5.6%)	1 (1.0%)	5 (5.1%)	6 (9.2%)	
Heroin, N (Col. %)	0 (0.0%)	0 (0.0%)	0 (0.0%)	0 (0.0%)	0 (0.0%)	
**Anthropomorphic variable** BMI^i^, mean (SD)	28.0 (4.9)	26.4 (5.7)	27.9 (4.8)	27.7 (4.8)	28.9 (4.9)	0.17
**Psychological variable** Depression, current CES-D > = 16/30^j^, N (Col. %)	195 (16.5%)	7 (14.9%)	57 (13.9%)	60 (15.8%)	71 (20.6%)	0.63

*BMI* body mass index, *CARDIA* coronary artery risk development in young adults study, *Col. %* column percentage, *N* number of participants, *+-SD* upper and lower limit of standard deviation.

^a^Self-reported cumulative exposure to cannabis joints in cannabis-years; 1 cannabis-year of exposure = 365 days of cannabis use (1 year × 365 days/y).

^b^P-values are from Kruskal-Wallis rank test for age, years of education, pack-years, drink-years, age started smoking, and BMI, from a χ2 test for race, study site, current smoking status, CES-D, and cumulative binge drinking categories, and from a Fisher exact test for cannabis and illicit drug use categories. Significant p-values marked in bold.

^c^By design, the CARDIA study sampled self-identified white men, white women, black men and black women in roughly equal numbers for participation in the study.

^d^Categories based on the answer to the question: “During the last 30 days, on how many days did you use marijuana?”

^e^Self-reported cumulative exposure to cigarettes in pack-years: 1 pack-year of exposure = 7300 cigarettes (1 year × 365 days/y × 1 pack/d × 20 cigarettes/pack).

^f^Cumulative alcohol use in drink-years: 1 drink-year is the total amount of ethanol consumed by a person who had 1 alcoholic drink per day for 1 year (1 drink-year = 17.24 ml of ethanol/drink x 1 drink/d x 365 days/y = 6292.6 ml of ethanol).

^g^Binge drinking days, defined as ≥5 drinks per day. If bingeing were to be constant over 30 years in one individual, 300 binge drinking days would correspond to 10 days of bingeing each year for 30 years.

^h^Current use, defined as any use within the last 30 days. Cocaine included all forms of cocaine, like crack, powder, free base; amphetamines included speed, uppers, and methamphetamines.

^i^Calculated as weight in kilograms divided by height in meters squared.

^j^Self-reported depression was measured every five years, starting at the Year 5 visit, on the Center for Epidemiologic Studies Depression scale (CES-D)0.18 A score of ≥16 was the cut-off for both sexes, indicating clinically significant depressive symptoms.

### Descriptive results

Mean hippocampal volume in never tobacco smoking participants overall was 3836 mm^3^ (SD 413). Mean hippocampal volume in never tobacco smoking participants reporting no cannabis use was 3800 mm^3^ (SD 425), and 3950 mm^3^ (SD 445) in participants reporting > 2 cannabis years (Table 2, Appendix Table 2 for combined dataset). Results were similar in ever tobacco smoking participants.

**Table 2:** Distribution of hippocampal volume, fractional anisotropy (FA) and cerebral blood flow (CBF) at Year 30 and cumulative exposure to cannabis in ‘cannabis-years’ among 648 CARDIA participants at the Year 30 MRI sub-study, excluding participants with a history of stroke or TIA.

NEVER TOBACCO SMOKING	All (340)	Never user	Ever Cannabis Use^a^		
			1 day to 0.5 cannabis years	0.5 to < 2 cannabis years	> 2 cannabis years
Volume Hippocampus (mm^3^), mean (SD)	3836 (413)	3800 (425)	3803 (421)	3919 (338)	3950 (445)
FA Fornix, mean (SD)	0.266 (0.035)	0.273 (0.034)	0.263 (0.036)	0.271 (0.036)	0.259 (0.031)
CBF Hippocampus (ml/100 g/min), mean (SD)	41.781 (9.941)	42.898 (10.397)	42.292 (8.773)	38.903 (10.091)	41.291 (13.332)
**EVER TOBACCO SMOKING**	**All (308)**	**Never user**	**Ever Cannabis Use**^**a**^		
			1 day to 0.5 cannabis years	0.5 to < 2 cannabis years	> 2 cannabis years
Volume Hippocampus (mm^3^), mean (SD)	3749 (397)	3652 (496)	3714 (390)	3789 (395)	3766 (384)
FA Fornix, mean (SD)	0.262 (0.036)	0.258 (0.023)	0.260 (0.036)	0.270 (0.036)	0.257 (0.038)
CBF Hippocampus (ml/100 g/min), mean (SD)	42.172 (8.770)	44.944 (6.835)	42.187 (9.112)	40.629 (7.705)	43.042 (9.879)

Stratified by ever tobacco smoking.

*FA* fractional anisotropy, *CBF* cerebral blood flow, *CARDIA* coronary artery risk development in young adults study, *N* number of participants; +-*SD* standard deviation.

aSelf-reported cumulative exposure to cannabis in cannabis-years; 1 cannabis-year of exposure = 365 days of cannabis use (1 year × 365 days/y).

### Main models

Mean hippocampal volume in never tobacco smoking participants as well as in ever tobacco smoking participants showed no significant differences between the cannabis exposure groups. For example, the coefficient of hippocampal volume in participants never smoking tobacco reporting more than 2 cannabis-years was −37.99 (95% CI −201.08 to +125.09) mm^3^, p-value for trend 0.3 (Table 3).

**Table 3:** Unadjusted and adjusted association between cumulative exposure to cannabis and hippocampal volume, fractional anisotropy (FA) and cerebral blood flow (CBF) at Year 30. 648 CARDIA participants at the Year 30 MRI sub-study excluding participants with a history of stroke or TIA.

NEVER TOBACCO SMOKING	Standardized difference in MRI measures (95% CI)^a^		
	Unadjusted	Demographic adjusted	Fully adjusted
**Hippocampus volume** (mm^3^)			
-Never user	Reference	Reference	Reference
-1 day to 0.5 cannabis years	−61.16 (−146.35–24.04)	−97.01 (−185.69 to −8.33)	−85.55 (−180.59–9.49)
-0.5 to < 2 cannabis years	−20.31 (−128.66–88.03)	−49.72 (−160.50–61.07)	−39.67 (−159.80–80.46)
->2 cannabis years	−29.15 (−162.25–103.96)	−85.42 (−227.52–56.69)	−37.99 (−201.08–125.09)
P-value for trend^b^ / R2	0.5/ 0.44	0.18/ 0.46	0.3/ 0.48
**Fornix FA**			
-Never user	Reference	Reference	Reference
-1 day to 0.5 cannabis years	−0.0098 (−0.0193 to −0.0002)	−0.0073 (−0.0168–0.0021)	−0.0052 (−0.0155–0.0051)
->2 cannabis years	−0.0144 (−0.0144–0.0097)	−0.0067 (−0.0217–0.0084)	−0.0013 (−0.0189–0.0164)
P-value for trend^b^/ R2	0.09/ 0.02	0.18/ 0.13	0.3/ 0.15
**Hippocampus CBF** (ml/100 g/min)			
-Never user	Reference	Reference	Reference
-1 day to 0.5 cannabis years	−0.61 (−4.03–2.82)	0.77 (−2.82–4.36)	0.49 (−3.39–4.36)
-0.5 to < 2 cannabis years	−3.99 (−8.44–0.45)	−1.46 (−6.05–3.12)	−1.43 (−6.49–3.62)
->2 cannabis years	−1.61 (−6.42–3.21)	1.29 (−3.81–6.39)	−0.40 (−6.60–5.80)
P-value for trend^b^/ R2	0.3/ 0.02	0.6/ 0.11	0.8/ 0.14
**EVER TOBACCO SMOKING**	**Standardized difference in MRI measures (95% CI)**^**a**^		
	Unadjusted	Demographic adjusted	Fully adjusted
**Hippocampus volume** (mm^3^)			
-Never user	Reference	Reference	Reference
-1 day to 0.5 cannabis years	−67.11 (−218.17–83.96)	−114.15 (−268.91–40.61)	−125.11 (−284.78–34.56)
-0.5 to < 2 cannabis years	−53.48 (−206.41–99.44)	−106.85 (−265.78–52.08)	−141.34 (−306.00–23.32)
->2 cannabis years	−105.82 (−260.84–49.20)	−139.86 (−300.75–21.03)	−152.20 (−323.60–19.20)
P-value for trend^b^/ R2	0.5/ 0.44	0.4/ 0.46	0.4/ 0.51
**Fornix FA**			
-Never user	Reference	Reference	Reference
-1 day to 0.5 cannabis years	0.0023 (−0.0158–0.0203)	−0.0012 (−0.0190–0.0165)	0.0010 (−0.0178–0.0198)
->2 cannabis years	−0.0011 (−0.0194–0.0172)	0.0004 (−0.0180–0.0188)	0.0052 (−0.0150–0.0255)
P-value for trend^b^/ R2	0.06/ 0.02	0.09/ 0.15	0.18/ 0.17
**Hippocampus CBF** (ml/100 g/min)			
-Never user	Reference	Reference	Reference
-1 day to 0.5 cannabis years	−2.76 (−8.10–2.58)	−0.33 (−5.43–4.78)	1.98(−3.13–7.08)
-0.5 to < 2 cannabis years	−4.32 (−9.74–1.11)	−1.30 (−6.63–4.03)	0.97(−4.48–6.41)
->2 cannabis years	−1.90 (−7.41–3.61)	2.37 (−3.13–7.86)	5.11 (−0.68–10.90)
P-value for trend^b^/ R2	0.4/ 0.02	0.2/ 0.24	0.10/ 0.41

Stratified by ever tobacco smoking.

*FA* fractional anisotropy, *CBF* cerebral blood flow, *CARDIA* coronary artery risk development in young adults study, *N* number of participants; 95% CI 95% confidence interval.

*Results from multivariable adjusted linear regression models. First unadjusted (only hippocampal volume adjusted for total intracerebral volume in unadjusted models), then adjusted for demographics (sex, race, age, education years, study center) and finally, current and cumulative alcohol use, cumulative cigarette smoking, self-reported lifetime illicit drug use (amphetamines, methamphetamines, cocaine, heroin), depression and BMI.

aLinear regression models determined the association between MRI scores and self-reported cumulative exposure to cannabis use. Negative standardized scores indicate smaller Volume, lower FA or lower CBF.

b P-values from a Wald test. All P-values two sided.

Overall, we found no significant association between cannabis use and hippocampal volume (p-value for trend 0.3), fractional anisotropy (FA, p-value for trend 0.3) or cerebral blood flow (CBF) (p-value for trend 0.8) in fully adjusted models in never tobacco smoking participants (Table 3, Appendix Table 3 for combined dataset).

### Analyses stratified by sex

In both tobacco user groups we found no significant association between cumulative cannabis use and hippocampal volume, fractional anisotropy or cerebral blood flow in analyses stratified by sex (Table 4). E.g. in the never tobacco smoking group for more than 2 years of cannabis use, men showed a mean hippocampal volume of −31.57 (95% CI −312.55–249.40) mm^3^ compared to never users, and women of −21.13 (95% CI −209.69–167.43) mm^3^ (Table 4, Appendix Table 4 for combined dataset).

**Table 4:** Association between cumulative exposure to cannabis and hippocampal volume, fractional anisotropy (FA) and cerebral blood flow (CBF) at Year 30, **stratified by sex, multivariable adjusted**.

Variable	NEVER TOBACCO SMOKING Standardized difference in MRI measures (95% CI)^a^		EVER TOBACCO SMOKING Standardized difference in MRI measures (95% CI)^a^	
	Women	Men	Women	Men
**Hippocampus volume** (mm^3^)				
-Never user	Reference	Reference	Reference	Reference
-1 day to 0.5 cannabis years	−25.66 (−122.13–70.82)	−137.17 (−304.84–30.49)	−160.03 (−346.02–25.96)	18.35 (−341.58–378.29)
-0.5 to < 2 cannabis years	57.60 (−77.46–192.65)	−158.19 (−356.04–39.66)	−174.60 (−371.99–22.79)	31.04 (−326.35–388.42)
->2 cannabis years	−21.13 (−209.69–167.43)	−31.57 (−312.55–249.40)	−124.94 (−341.89–92.01)	−94.22 (−449.17–260.73)
P-value for trend^a^/ R2	0.5/ 0.5	0.3/ 0.4	0.3/ 0.51	0.4/ 0.45
**Fornix FA**				
-Never user	Reference	Reference	Reference	Reference
-1 day to 0.5 cannabis years	0.0005 (−0.0125–0.0134)	−0.0084 (−0.0247–0.0079)	0.0059 (−0.0153–0.0272)	0.0137 (−0.0252–0.0526)
->2 cannabis years	−0.0005 (−0.0253–0.0242)	−0.0090 (−0.0362–0.0182)	0.0091 (−0.0156–0.0338)	0.0154 (−0.0229–0.0537)
P-value for trend^b^/ R2	0.6/ 0.22	0.7/ 0.15	0.5/ 0.31	0.7/ 0.27
**Hippocampus CBF** (ml/100 g/min)				
-Never user	Reference	Reference	Reference	Reference
-1 day to 0.5 cannabis years	1.26 (−4.82–7.34)	−0.98 (−6.11–4.16)	7.27 (0.60–13.93)	−4.15 (−16.87–8.57)
-0.5 to < 2 cannabis years	−2.28 (−10.74–6.17)	−1.54 (−7.98–4.90)	5.08 (−1.84–11.99)	−5.70 (−18.21–6.82)
->2 cannabis years	2.06 (−7.91–12.03)	−4.18 (−12.50 to)	9.96 (1.62–18.29)	−0.96 (−13.33–11.42)
P-value for trend^b^/ R2	0.7/ 0.13	0.8/ 0.28	0.09/ 0.45	0.3/ 0.48

648 CARDIA participants at the Year 30 MRI sub-study excluding participants with a history of stroke or TIA.

**Stratified by ever tobacco smoking**.

*FA* fractional anisotropy, *CBF* cerebral blood flow, *CARDIA* coronary artery risk development in young adults study, *N* number of participants; 95% CI 95% confidence interval.

^a^P-values from a Wald test. All P-values two sided.

### Sensitivity analyses

**Fig. 1:**
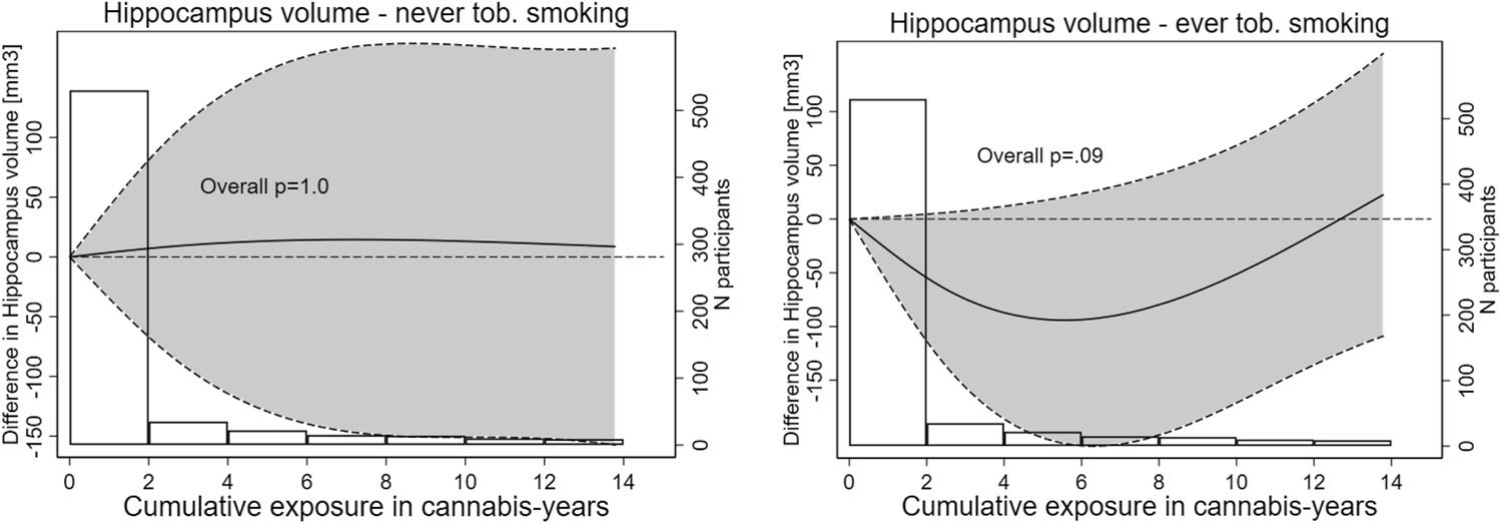
Association between cumulative cannabis use (modelled flexibly) and hippocampal volume. 648 CARDIA participants at the Year 30 MRI sub-study excluding participants with a history of stroke or TIA. **Left: never tobacco smoking, right: ever tobacco smoking**. Reference (0) corresponds to the multivariable adjusted hippocampal volume in never cannabis users, and is compared to flexibly modelled cumulative cannabis exposure in ever cannabis users. Results from multivariable adjusted linear regression models, using splines with three knots. Adjusted for total intracerebral volume, demographics (sex, race, age, education years, study center), current and cumulative alcohol use, cumulative cigarette smoking, self-reported lifetime illicit drug use (amphetamines, methamphetamines, cocaine, heroin), depression and BMI. Cumulative exposure to cannabis expressed in cannabis-years, with 1 cannabis-year of exposure equivalent to 365 days of cannabis use.

(1) When testing the left and right hemisphere separately, we could not find an association between cannabis use hippocampal volume, integrity or cerebral blood flow (Appendix Table 5). Results remained unchanged (2) after adjusting for the mirror star tracing test at Year 2, (3) after adjusting for additional cardiovascular risk factor measurements, or (4) when including participants with a history of stroke or TIA (Appendix Tables 5 and 6). (5) We found no significant association between cumulative cannabis use categories and hippocampal outcomes in current cannabis users only and in current cannabis abstainers (no use in past 30 days, Appendix Table 6). (6) Results were similar in models with cumulative cannabis use modeled as non-linear continuous variable (see Fig. 1 including information on number of participants in slices of 2 cannabis-years of exposure), Appendix Fig. 1 and 2). All the above-mentioned findings apply to both the never and the ever tobacco using group.

### Exploratory secondary analyses

We found no significant association between cannabis use and volume of the amygdala or prefrontal cortex (PFC; see Appendix Tables 7 and 8).

## Discussion

Self-reported cumulative cannabis use was not associated with hippocampal outcomes in multivariable adjusted models in this cohort of middle-aged adults, stratified by ever tobacco smoking. We found no significant association between cannabis and hippocampal volume, structural integrity or blood flow overall and in analyses stratified by sex or by hippocampal side. These results were robust in several sensitivity analyses.

### Volumetric analyses

Previous studies examining the relationship between cannabis use and hippocampal volumes suggested that heavy cannabis users tend to have smaller hippocampal volumes compared to non-users [9–11, 13–15].

One of the largest studies on cannabis and brain volume, involving 86 participants with long-term cannabis use, found lower cognitive reserve as well as smaller hippocampal volume in long-term cannabis users [12]. Our study could not confirm an overall association between cannabis use and smaller hippocampal volumes. One difference may lie in the definition of cannabis use. In the referenced study, cannabis users were categorized into different groups: long-term users, midlife recreational users, quitters and never users. However, the study did not quantify cumulative exposure. In contrast, our study utilized cumulative exposure as the primary criterion for group classification, allowing for a clearer examination of associations between different levels of cannabis exposure and hippocampus outcomes. Furthermore, this cohort had almost exclusively white participants (93%) while in our study ethnicity was more evenly distributed (60% white). Our participant cohort also consisted of individuals with an older age profile (56 years vs. 45 years).

Other studies reported no association between cumulative cannabis exposure and hippocampal outcomes: for example, a large twin-study including 158 young adults using cannabis least weekly that controlled for various substance use variables found that cannabis use was unrelated to any ROI [55]. One meta-analysis found that cannabis dosage and duration of use were not associated with hippocampal volumes. This suggests that cannabis dosage and duration of use does not mainly drive the volumetric reduction in cannabis users [56, 57]. It is thus plausible that cannabis and cognition are associated independently of hippocampal structure, and future studies should study further neuronal mechanisms and social determinants of health associated with cognition in cannabis users. Observed differences in previous cross-sectional studies may reflect earlier developmental or co-morbid factors rather than ongoing cannabis-related neurodegeneration [57]. Also, some authors have suggested cannabis dependency was associated with brain regions, but not cumulative use per se (see Appendix) [14, 58]. This highlights the importance of examining differences that predate cannabis use and to adjust for relevant sociologic confounders. Studying social determinants of cannabis use would possibly help identifying measures to tackle cannabis associated pathologies.

As mentioned above, we found an association between cumulative use of cannabis and worse verbal memory in men but not in women in a previous investigation [6]. The findings of this study do not provide a brain morphological explanation for this association. Instead, they, along with previous research, suggest that attributing cannabis-related cognitive deficits solely to smaller hippocampal volume through a neurodegenerative process may be overly simplistic. In addition to the hippocampus, other cannabinoid receptor CB1-rich brain regions, including those involved in reward and motivation, may play a role [12]. For example, alterations in the connectivity and function between the hippocampus and the orbitofrontal cortex (OFC, part of the PFC) have been shown to be associated with regular cannabis use and other substance use disorders, indicating a common neurobiological signature. In our exploratory analyses, we found cannabis use and the prefrontal cortex (PFC) outcomes were not associated. Moreover, factors like stress, socioeconomic conditions, comorbid substance use and individual differences in vulnerability may further contribute to these neural changes and cognitive outcomes, making it essential to consider a broader range of influences beyond hippocampal volume alone [56].

### DTI

Previous studies using diffuse tensor imaging (DTI) reported both intact [16] and altered [17, 18] white matter. The most consistent differences between cannabis users and controls included lower fractional anisotropy in the arcuate/superior longitudinal fasciculus and in the corpus callosum [59]. Two studies found that cannabis users had impaired connectivity in areas in and near the right hippocampus (possibly the fornix) [18, 19]. However, both studies have some limitations. Participants in one study exhibited more depressive symptoms than those in our cohort [18]. Since depression is linked to changes in brain microstructure, this could have influenced the results. Additionally, that study recruited participants through advertisements, making it vulnerable to selection bias [18].. In a subsequent study by Yücel et al [60]., following the second study mentioned above [19], no significant differences in hippocampal FA values were found between cannabis users and non-users. This later study included a larger sample size and improved controls for both other drug use and sociodemographic factors [60]. Another recent study controlling for mental health and illicit drug use found that white matter microstructure did not differ between cannabis users and controls and did not covary with recent cannabis use, dependence severity, or duration of use [61]. Our findings align with those later findings as we could not report an association between cannabis use and altered FA.

### CBF

While one previous study [21] found hyperactivity in the hippocampus, most other studies found hypoactivity in hippocampal areas during cognitive tasks in cannabis users [22–24]. One study performed in resting state, found that cannabis users exhibited higher cerebral blood flow (CBF) in the right pallidum/putamen as well as higher regional CBF of the right superior frontal cortex compared to nonusers. The study did not report any findings regarding CBF in the hippocampus [62]. In our study, we could not find associations between cumulative cannabis use and altered CBF in the hippocampus. However, it is to keep in mind that we measured CBF in resting state and not during cognitive tasks. Studies including even more sensitive MRI measures including task-based MRI or subfield segmentation are warranted.

### Limitations

Our study has several limitations. The CARDIA study was primarily designed to examine the factors that contribute to developing cardiovascular disease. While it is the largest study in the U.S. to collect information on cannabis use on multiple visits over 30 years, allowing us to compute cumulative exposure to cannabis more precisely then when queried on a single occasion in middle-age, substance use and its potential adverse effects on brain structure were not CARDIA’s primary focus, and heavy users are probably underrepresented in this study. We believe the strength of the CARDIA is on testing the potential effect of cumulative exposure to cannabis and not acute exposure, as acute cannabis exposure is likely to lead to very dynamic effects in the brain and we would need much more detailed information on the exact timing, dose and administration route of the last exposures prior to the MRI exam, which we do not have. The CARDIA questionnaire also did not inquire about the modality of use of cannabis (e.g. smoked or ingested) or its type (e.g. THC content). We constructed cannabis exposure measurement from self-reported information collected periodically over 30 years; these assessments of self-reported cannabis use were up to 5 years apart. We extrapolated self-reported past month cannabis use up to a 5-year period, assuming an invariant use of cannabis, as has been done previously [31, 36, 47, 63]. Cannabis years are thus an approximation of true cumulative cannabis use. Still, this is the computation approach allowing for the highest granularity of cannabis exposure in a cohort study over 30 years. Also, although CARDIA was drawn from the general population and included Black and White participants, generalizability to other ethnicities is limited. Further, MRI was performed at a single time point in midlife, so causal inference regarding cumulative exposure and structural change is limited. Also, MRI precision limited findings, as our point estimates, ranging from 38–85 mm^3^, are close to the threshold of what can be distinguished from measurement variability [64]. Still, previous studies highlighted that differences in hippocampus in this magnitude are very likely to be attributable to normal inter-individual variability or a fraction of age‑related change [65]. Also, in our cohort the Cerebral Blood Flow analyses were performed in a resting state. However, in most previous studies, CBF was tested during cognitive tasks. Therefore, the interpretation and comparability of our results are limited. Finally, we lacked data on cannabis dependency, which might be associated with cannabis use and cognition via more complex pathways than identified when studying isolated cerebral regions.

### Conclusion

In our large biracial cohort study drawn from the general population, and following participants over 30 years, and assessing cannabis use every 2–5 years, we found no significant association between self-reported cumulative cannabis exposure and hippocampal volume, integrity or blood flow in middle age. These results were robust in the overall sample and stratified by ever tobacco smoking. The differences in memory function in recreational cannabis users from the general population are likely not attributable to the hippocampal structure or integrity only. Future studies should assess further neuronal mechanisms (task-based fMRI or subfield segmentation) and social determinants of health associated with cognition in cannabis users.

## Supplementary information

Supplementary Material

## Data Availability

Data is available upon request.
