# Supplementary material for: Cumulative exposure to cannabis and hippocampus MRI in middle age: results from the coronary artery risk development in young adults (CARDIA) study
Source: Transl Psychiatry. 2026 May 19;16:354. doi: 10.1038/s41398-026-04096-1 (PMC13350708; doi:10.1038/s41398-026-04096-1)
Supplement: Supplementary file 1 — Supplementary Material [file 41398_2026_4096_MOESM1_ESM.docx]

**Supplementary Material**

**Cumulative Exposure to Cannabis and Hippocampus MRI in Middle Age: results from the Coronary Artery Risk Development in Young Adults (CARDIA) study.**

Barbara Schilling, MA^1^, Baptiste Pasquier, MD^1^, Martine El Bejjani, PhD ^2^, Jared Reis, PhD^3^, Jamal S. Rana, MD, PhD^4^, Kali Tal, PhD^1^, Lenore J. Launer, PhD^5^, Stéphanie Baggio, PhD^1,6^, Stephen Sidney, MD^7^, Nick Bryan, MD, PhD^8^, Kristine Yaffe, MD^9^, Reto Auer MD, MAS ^1,10^ and Julian Jakob, MD, PhD^1,11^

^1^ Institute of Primary Health Care (BIHAM), University of Bern, Bern, Switzerland, ^2^ American University of Beirut, Beirut, Lebanon, ^3^ National Heart, Lung, and Blood Institute, Bethesda, MD,USA, ^4^ Kaiser Permanente Northern California, Department of Cardiology and Division of Research, Oakland, CA, USA, ^5^ National Institute on Aging, Bethesda, MD, USA, ^6^ Institute of Psychology, University of Lausanne, Lausanne, Switzerland, ^7^ Kaiser Permanente Division of Research, Oakland, CA, USA, ^8^ University of Pennsylvania, Philadelphia, PA, USA, ^9^ University of California San Francisco, San Francisco, CA, USA, ^10^ Center for Primary Care and Public Health (Unisanté), University of Lausanne, Lausanne, Switzerland, ^11^ Department of Paediatrics, University Hospital Bern, Inselspital, Bern, Switzerland

**Corresponding author**Julian Jakob, MD PhD
Institute of Primary Health Care (BIHAM)
University of Bern
Mittelstrasse 43, CH-3012 Bern, Switzerland
Email: [julian.jakob@unibe.ch](mailto:julian.jakob@unibe.ch)
Phone: +41 31 684 58 64

**Content**

[eMethods 4](#_Toc221096789)

[**Measurements** 4](#_Toc221096790)

[Cannabis use 4](#_Toc221096791)

[Cigarette smoking exposure 5](#_Toc221096792)

[Alcohol exposure 5](#_Toc221096793)

[Other illicit drug exposure 6](#_Toc221096794)

[Cardiovascular risk factors 6](#_Toc221096795)

[Psychosocial measures 6](#_Toc221096796)

[Mirror star tracing test 6](#_Toc221096797)

[**Statistical analysis** 7](#_Toc221096798)

[Mirror star tracing test 7](#_Toc221096799)

[**STROBE Statement – Filled Checklist (pages refer to the text document only, with tables situated after references)** 9](#_Toc221096800)

[**eTables and eFigures** 12](#_Toc221096801)

[**Appendix Table 1:** Characteristics of 648 CARDIA participants with participation on the Year 30 MRI sub-study excluding participants with a history of stroke or TIA 12](#_Toc221096802)

[**Appendix Table 2**: Distribution of hippocampal volume, fractional anisotropy (FA) and cerebral blood flow (CBF) at Year 30 and cumulative exposure to cannabis in ‘cannabis-years’ among 648 CARDIA participants at the Year 30 MRI sub-study, excluding participants with a history of stroke or TIA. 15](#_Toc221096803)

[**Appendix Table 3:** Unadjusted and adjusted association between cumulative exposure to cannabis and hippocampal volume, fractional anisotropy (FA) and cerebral blood flow (CBF) at Year 30. 648 CARDIA participants at the Year 30 MRI sub-study excluding participants with a history of stroke or TIA. 16](#_Toc221096804)

[**Appendix Table 4.** Association between cumulative exposure to cannabis and hippocampal volume, fractional anisotropy (FA) and cerebral blood flow (CBF) at Year 30, **stratified by sex, multivariable adjusted**. 648 CARDIA participants at the Year 30 MRI sub-study excluding participants with a history of stroke or TIA. 17](#_Toc221096805)

[**Appendix Table 5:** Sensitivity analysis: Fully adjusted association between cumulative exposure to cannabis and **hippocampal** outcomes: **left and right hemisphere separatel**y, **adjusting for mirror star tracing test**, and **adjusting for additional CVRF**. 648 CARDIA participants with participation at the Year 30 MRI sub-study excluding participants with a history of stroke or TIA. **Stratified by ever tobacco smoking.** 18](#_Toc221096806)

[**Appendix Table 6:** Sensitivity analysis: Fully adjusted association between cumulative exposure to cannabis and hippocampal outcomes: **including participants with history of stroke or transient ischemic attack (TIA)**; only participants with **ongoing current** (in past 30 days) cannabis use; or **without current** cannabis use at their year 30 visit, separately. **Stratified by ever tobacco smoking.** 20](#_Toc221096807)

[**Appendix Table 7:** Exploratory Analysis: **Distribution** of volume of the Amygdala and of the Prefrontal Cortex (PFC) at Year 30 and cumulative exposure to cannabis in ‘cannabis-years’^a^ among 648 CARDIA participants with participation in the Year 30 MRI sub-study excluding participants with a history of stroke or TIA. **Stratified by ever tobacco smoking.** 22](#_Toc221096808)

[**Appendix Table 8:** Exploratory Analysis: Fully adjusted association between volume of the **Amygdala** or the **Prefrontal Cortex** (PFC) and **cumulative exposure to cannabis** at Year 30. 648 CARDIA participants with participation in the Year 30 MRI sub-study excluding participants with a history of stroke or TIA. Stratified by ever tobacco smoking. **Stratified by ever tobacco smoking.** 23](#_Toc221096809)

[**Appendix Figure 1:** Association between cumulative cannabis use (modelled flexibly) and fractional anisotropy (FA) at Year 30. 648 CARDIA participants at the Year 30 MRI sub-study excluding participants with a history of stroke or TIA. **Left: never tobacco smoking, right: ever tobacco smoking.** 24](#_Toc221096810)

[**Appendix Figure 2**: Association between cumulative cannabis use (modelled flexibly) and cerebral blood flow (CBF) at Year 30. 648 CARDIA participants at the Year 30 MRI sub-study excluding participants with a history of stroke or TIA. **Left: never tobacco smoking, right: ever tobacco smoking.** 25](#_Toc221096811)

[**eDiscussion** 25](#_Toc221096812)

[**eReferences** 27](#_Toc221096813)

# eMethods

## **Measurements**

### Cannabis use

We present below an example of one participant included in the study illustrating the method for computing cannabis-years more fully and how we applied linear imputation (Box 1). This is the same method of imputation used in a previous publication by our research group.[1]

Box 1. Example of computing cannabis-years for one participant:

| visit | mj30d_ | mj30d_imp | domj | life_ | yomj_max |
| --- | --- | --- | --- | --- | --- |
| 0 | 15 | 15 | 300 | 100 to 499 times | 0.82 |
| 1 | . | 15 | 482 | . | 1.32 |
| 2 | 20 | 20 | 725 | 100 to 499 times | 1.99 |
| 3 | . | 20 | 968 | . | 2.65 |
| 4 | . | 4 | 1017 | . | 2.79 |
| 5 | 4 | 4 | 1066 | 100 to 499 times | 2.92 |
| 6 | . | 4 | 1115 | . | 3.05 |
| 7 | 20 | 20 | 1358 | 100 to 499 times | 3.72 |
| 8 | . | 20 | 1601 | . | 4.39 |
| 9 | . | 20 | 1844 | . | 5.05 |
| 10 | 20 | 20 | 2087 | 500 to 1000 times | 5.72 |
| 11 | . | 20 | 2330 | . | 6.38 |
| 12 | . | 20 | 2573 | . | 7.05 |
| 13 | . | 10 | 2695 | . | 7.38 |
| 14 | . | 10 | 2817 | . | 7.72 |
| 15 | 10 | 10 | 2939 | 500 to 1000 times | 8.05 |
| 16 | . | 10 | 3061 | . | 8.39 |
| 17 | . | 10 | 3183 | . | 8.72 |
| 18 | . | 1 | 3195 | . | 8.75 |
| 19 | . | 1 | 3207 | . | 8.79 |
|  |  |  |  |  |  |
| 20 | 1 | 1 | 3219 | 100 to 499 times | 8.82 |

Visit: visit year; mj30d_: Self-reported days of using cannabis during the month before the visit (“During the last 30 days, on how many days did you use cannabis?”), mj30d_imp: imputed mj30d_ variable; domj: computed cumulative days of cannabis use, life_: categorical variable on self-reported lifetime use of cannabis queried at each visit (“About how many times in your lifetime have you used marijuana?”), yomj_max: computed cumulative years of cannabis use (domj/365).

According to example 1, the participant reported having used cannabis 100 to 499 times during the lifetime (categorical variable life_) at the baseline examination (visit 0). This was used to estimate the exposure prior to the first examination (domj at visit 0=300, where domj signifies “days of cannabis”).
At this baseline examination, the participant reported using cannabis 15 days per month (mj30d_). Multiplied by 12.17 months (365/30), we estimated that this participant used cannabis 182 days in the first year after the first exam (15 * 12.17). The number of days of cannabis use in the month before the baseline examination was imputed forward at year 1 (mj30d_imp). At year 2, the participant reported using cannabis 20 days per month; at year 5, he/she reported 4 days per month. These numbers were imputed backwards and forwards; when there were an uneven number of intervals (preventing us from evenly splitting the imputation based on the prior value as opposed to the post-value), the exposure at the prior interval was favored arbitrarily. Participants then accrued lifetime days of cannabis use over follow-up. The cumulative number of cannabis-years over lifetime is presented in the last column (yomj_max). For this participant, the cumulative number of cannabis years was 8.82, corresponding to 3,219 estimated days of cannabis use, which contrasts largely with the lifetime estimate the participant reported when asked about how many times he/she used cannabis (recall bias). We found no change in the estimates when using alternate methods for imputing missing values such as using the mean number of days of cannabis use between two examinations with data on this variable.

### Cigarette smoking exposure

Detailed cigarette smoking behavior was evaluated during each in-person CARDIA visit by responses to an interviewer-administered questionnaire.[2] Participants were also asked the number of cigarettes smoked daily at yearly contact over the phone between CARDIA visits. Current smoking was defined as smoking one cigarette or more per day. At baseline, participants were asked the number of years they had smoked in total, the age at which they started smoking regularly (“How old were you when you started smoking cigarettes regularly?”) and years since cessation (“How many years ago did you stop?”), if appropriate. These data were used to estimate cumulative lifetime exposure to cigarettes in terms of pack-years, with 1 pack-year of exposure equivalent to 7300 cigarettes (1 year × 365 days/y × 1 pack/d × 20 cigarettes/pack).[1]

### Alcohol exposure

Alcohol consumption was measured during each CARDIA visit. We estimated lifetime alcohol consumption in “drink-years,” defining 1 drink-year as the amount of alcohol consumed in 1 year by a person consuming 1 drink/day, as previously reported.[3] Categories of alcohol consumption (abstinent, light, heavy) were based on the sex-specific weekly maximum drinking limits published by the National Institute on Alcohol Abuse and Alcoholism [for men >14 (women >7) standard drinks/week or >4 (>3) drinks/day].[4] Acute heavy exposure to alcohol (bingeing) at the Year 20 visit was defined as reporting 5 or more drinks to the following question, “During the past 24 hours, how many drinks have you had?” Information on bingeing was elicited in a separate question and allowed us to estimate the cumulative number of binge drinking days. Alcohol consumption was measured during each CARDIA visit. Participants were asked, “Did you drink any alcoholic beverages in the past year?” and three follow-up questions regarding how many drinks of wine, beer, and liquor they usually consumed per week. Assuming that one drink of beer, wine, or liquor contains 16.7 ml, 17.0 ml, or 19.1 ml of ethanol, respectively (per CARDIA protocol), we estimated total ethanol consumption per week in milliliters of ethanol and divided it by 17.24 ml of ethanol per average drink to estimate the usual number of drinks per week that each participant reported at each visit. We estimated lifetime alcohol consumption in “drink-years,” defining 1 drink-year as the amount of alcohol consumed in 1 year by a person consuming 1 drink/day (365 days/year ×17.24 ml of alcohol/day = 6,293 ml of alcohol). Binge drinking at the Year 20 visit was assessed directly by asking participants: “During the past 30 days, on how many days did you have five or more drinks on the same occasion?” For the other visits, we computed bingeing as follows: At each visit, participants were asked: “In the past month what is the largest number of drinks you had in one day?” At baseline, participants were additionally asked: “How many days in the past month did you have about (number of drinks answered in the previous question) drinks?” We used the number of days participants reported having 5 or more drinks for these visits. For binge drinking events at the Year 7 visit, we used the closest available information about the number of days patients reported having 5 or more drinks if they reported having such a use in one day within the last month at the Year 7 visit.

### Other illicit drug exposure

Other illicit substances queried included cocaine (including other forms of cocaine such as crack, powder, free base), amphetamines (speed, uppers, methamphetamines) and heroin.[5] Participants were asked: “Have you ever used (substance)?”; “During the last 30 days, on how many days did you use (substance)?” and “How many times in your lifetime have you used (substance)?” The number of days on cocaine, crack, speed, methamphetamines and heroin over the study duration was computed using current exposure at each visit and replaced by lifetime exposure when the latter was higher.

### Cardiovascular risk factors

Blood pressure was measured on the right arm with a Hawksley random zero sphygmomanometer (WA Baum Company, Copaigue, NY) by trained and certified technicians using standardized methods after the participant had rested for 5 minutes at baseline and Year 7, and at Year 20, a digital blood pressure monitor (Omron HEM-907XL; Online Fitness, Santa Monica, CA) was used.
Three measurements were obtained at 1-minute intervals. The average of the second and third measurements was used in analyses. Fasting total cholesterol and triglycerides were measured enzymatically at baseline, Years 7, and 20 by the Northwest Lipid Research Laboratory at the University of Washington (Seattle, WA). For all visits, HDL cholesterol was determined by dextran sulfate –magnesium precipitation on the Abbot Spectrum, and LDL cholesterol was calculated using the Friedewald equation. At each visit, weight and height were measured, and body mass index (BMI) was calculated as weight in kilograms divided by height in meters squared. We built a cumulative measure of each cardiovascular risk factor (BMI, blood cholesterol, blood triglycerides, blood pressure) by calculating the area under the curve from year 0 through year 30.

### Psychosocial measures

Depressive symptoms have been associated with cannabis use and cognitive impairment[6]. In the main model we adjusted for the self-reported depression using the Center for Epidemiologic Studies Depression scale (CES-D). Self-reported depression was measured every five years, starting at the Year 5 visit. A score of ≥16 was the cut-off for both sexes, indicating clinically significant depressive symptoms.

### Mirror star tracing test

At Year 2, the mirror star tracing test was conducted to elicit reactive blood pressure. In the mirror star-tracing test, participants had to trace the outline of a star from a reversed image displayed in a mirror while staying within narrow limits. Study participants were instructed to draw stars as quickly as possible with the fewest possible errors. If they moved out of the limits of the star, an error was scored. Total stars completed and total numbers of errors over three minutes were recorded. Although initially intended as a stressor to measure blood pressure reactivity and not as a cognitive test, some have suggested that the mirror star tracing test measures aspects of executive function.[3]

**MRI (based on [7])**

Parameters of interest were estimated as follows: From the sagittal 3D T1 sequence (Plane Sagittal Coil 12channel File name 3D T1 MPRAGE: Tr 1900 Te 2.89 Fov 250mm, thickness 1mm slices 176 slices, Base Res 255, Phase res 100%, Matrix 256X256 NSA 1 TI 900 ms Pixel BW 170hz. ETL = 1 Flip = 9), we estimated total intracranial volume (TICV), (a measure of head size) as the sum of gray matter (GM),white matter (WM) and cerebral spinal fluid (CSF) volumes, and total brain tissue volume (TBV) as the sum of GM and WM volumes). We estimated abnormal white matter tissue from the sagittal 3D FLAIR (Plane Sagittal Coil 12channel File name 3D FLAIR: Tr 6000 Te 160 Fov 250mm (fov phase = 85%), thickness 1 mm slices 160 slices, Base Res 202, Phase res 91%, Matrix 258 X 221 NSA 1 TI = 2200 ms Pixel BW 930, ETL 203)), T1 and T2 (Plane Sagittal Coil 12 channel Psd File name 3D T2: Tr 3200 Te 409 Fov 250 (fov phase = 80%), thickness 1mm slices 176, Base Res = 246, Phase res = 80%, Matrix 258x256 NSA 1 Center Freq. water ETL 141 Flip 120 Pixel BW 750) sequences. Brain microstructural tissue integrity and organization were estimated from axial Diffusion Tensor Images (DTI); Plane Axial Coil 12-channel Psd File name ep2d diff MDDW: TR 7300 TE 84 Fov 245 Thickness 2.2mm distance factor 0% Diff Directions- 33 Concatenations = 1 number sl = 64 Flip 90 Matrix 128x128 NSA 1 Center Freq. water Phase FOV = 100 Pixel BW = 1860 diff mode = Free Phase part fourier = 7/8 Echo spacing = .59 diff weighting = 1 Accel factor = 3 EPI factor = 112 Base Res 112 Phase res 100%; 2 times). Here we report on the WM-DTI—derived fractional anisotropy (FA) measure, which ranges from 0 to 1 and estimates the degree (or uniformity) to which water diffuses along the direction of myelinated tracks in the white matter. Zero indicates equal probability of diffusion in all directions (i.e. there is no structural restriction to the flow of molecules), and a ‘1’ indicates the diffusion occurs along one axis (i.e., the WM tract). Cerebral brain perfusion (CBF; volume of flow per unit brain mass per unit time (mL/100g/min)) was measured with an axial pseudo-Continuous Arterial Spin Labeling (pCASL) technique (Plane Axial Coil 12 channel Psd File name pCASL: Tr 4000 Te 11 Fov 220 mm, Concatenations 1 number sl 20, Base Res 64, Phase res = 100%, thickness 5, distance factor 20%, Center Freq. water, Matrix 64 x 64 NSA 1 Fat suppression = ON Flip = 90 Echo spacing = .47). Here we present the estimate for the GM as it is more reliably obtained than measures in WM. Per participant, completion rates for the sequences ranged from 95.9% (pCASL) to 100% (T1, PD/T2 and FLAIR sequences). See full information here: [7].

## **Statistical analysis**

### Mirror star tracing test

We tested the association between mirror star tracing and hippocampal volume by including the number of errors and stars traced as covariables in the main multivariable logistical regression models of the association between cumulative cannabis use and CF stratified by sex. Covariables were the same as those included in the main model testing the association between cumulative cannabis use of CF: age, race, study site, education, cigarette smoking, alcohol, illicit drug use, BMI, depression at the Year 30 visit. Drink-years of alcohol, binge-drinking episodes, tobacco pack-years, and BMI, were all modelled as 3-knot restricted cubic splines. Each time-dependent covariable included data up to the years of MRI assessment (at the Year 2 and Year 30 visits).

# **STROBE Statement – Filled Checklist (pages refer to the text document only, with tables situated after references)**

|  | Item no | Recommendation | Done | Page |
| --- | --- | --- | --- | --- |
| **Title and abstract** | 1 | (*a*) Indicate the study’s design with a commonly used term in the title or the abstract | √ | 3 |
|  |  | (*b*) Provide in the abstract an informative and balanced summary of what was done and what was found | √ | 3 |
| Introduction | | |  |  |
| Background/ rationale | 2 | Explain the scientific background and rationale for the investigation being reported | √ | 5 |
| Objectives | 3 | State specific objectives, including any prespecified hypotheses | √ | 5 |
| Methods | | |  |  |
| Study design | 4 | Present key elements of study design early in the paper | √ | 3 |
| Setting | 5 | Describe the setting, locations, and relevant dates, including periods of recruitment, exposure, follow-up, and data collection | √ | 6, see also references listed in the methods |
| Participants | 6 | (*a*) Give the eligibility criteria, and the sources and methods of selection of participants. Describe methods of follow-up | √ | 6, see also references listed in the methods and Supplement |
|  |  | (*b*) For matched studies, give matching criteria and number of exposed and unexposed | - | - |
| Variables | 7 | Clearly define all outcomes, exposures, predictors, potential confounders, and effect modifiers. Give diagnostic criteria, if applicable | √ | 6-8, Table 1, and Supplement |
| Data sources/ measurement | 8* | For each variable of interest, give sources of data and details of methods of assessment (measurement). Describe comparability of assessment methods if there is more than one group | √ | 7, 8 and Supplement |
| Bias | 9 | Describe any efforts to address potential sources of bias | √ | 8 and Supplement |
| Study size | 10 | Explain how the study size was arrived at | √ | 9 |
| Quantitative variables | 11 | Explain how quantitative variables were handled in the analyses. If applicable, describe which groupings were chosen and why | √ | 7-8 and Supplement |
| Statistical methods | 12 | (*a*) Describe all statistical methods, including those used to control for confounding | √ | 6-8 and Supplement |
|  |  | (*b*) Describe any methods used to examine subgroups and interactions | √ | 6-8 |
|  |  | (*c*) Explain how missing data were addressed | √ | 8 and Supplement |
|  |  | (*d*) If applicable, explain how loss to follow-up was addressed | √ | 8 and Supplement |
|  |  | (*e*) Describe any sensitivity analyses | √ | 8 |
| Results | | |  |  |
| Participants | 13* | (a) Report numbers of individuals at each stage of study—eg numbers potentially eligible, examined for eligibility, confirmed eligible, included in the study, completing follow-up, and analysed | √ | 9 |
|  |  | (b) Give reasons for non-participation at each stage | ? | n/a |
|  |  | (c) Consider use of a flow diagram | - | - |
| Descriptive data | 14* | (a) Give characteristics of study participants (eg demographic, clinical, social) and information on exposures and potential confounders | √ | 9, Table 1 and Supplement |
|  |  | (b) Indicate number of participants with missing data for each variable of interest | - | - |
|  |  | (c) Summarise follow-up time (eg, average and total amount) | √ | 6 and Supplement |
| Outcome data | 15* | Report numbers of outcome events or summary measures over time | √ | 9 |
| Main results | 16 | (*a*) Give unadjusted estimates and, if applicable, confounder-adjusted estimates and their precision (eg, 95% confidence interval). Make clear which confounders were adjusted for and why they were included | √ | 7-8, Table 3 |
|  |  | (*b*) Report category boundaries when continuous variables were categorized | √ | 6-8 Table 1-4 |
|  |  | (*c*) If relevant, consider translating estimates of relative risk into absolute risk for a meaningful time period | - | - |
| Other analyses | 17 | Report other analyses done—eg analyses of subgroups and interactions, and sensitivity analyses | √ | 8-10, eTable 1-3 |
| Discussion | | |  |  |
| Key results | 18 | Summarise key results with reference to study objectives |  | 9,11, Table 2 - 4, |
| Limitations | 19 | Discuss limitations of the study, taking into account sources of potential bias or imprecision. Discuss both direction and magnitude of any potential bias | √ | 12 |
| Interpretation | 20 | Give a cautious overall interpretation of results considering objectives, limitations, multiplicity of analyses, results from similar studies, and other relevant evidence | √ | 9-12 |
| Generalisability | 21 | Discuss the generalisability (external validity) of the study results | √ | 11-12 |
| Other information | | |  |  |
| Funding | 22 | Give the source of funding and the role of the funders for the present study and, if applicable, for the original study on which the present article is based | √ | 1 |

*Give information separately for exposed and unexposed groups.

**Note:** An Explanation and Elaboration article discusses each checklist item and gives methodological background and published examples of transparent reporting. The STROBE checklist is best used in conjunction with this article (freely available on the Web sites of PLoS Medicine at http://www.plosmedicine.org/, Annals of Internal Medicine at http://www.annals.org/, and Epidemiology at http://www.epidem.com/). Information on the STROBE Initiative is available at <http://www.strobe-statement.org>.

# **eTables and eFigures**

### **Appendix Table 1:** Characteristics of 648 CARDIA participants with participation on the Year 30 MRI sub-study excluding participants with a history of stroke or TIA

| **Variable** | **All** | **Never** | **Ever Cannabis Use^a^** | | | **p-value^b^** |
| --- | --- | --- | --- | --- | --- | --- |
|  |  |  | 1 day to 0.5 cannabis years | 0.5 to < 2 cannabis years | > 2 cannabis years |  |
| N (%) | 648 (100%) | 93 (14%) | 280 (43%) | 157 (24%) | 118 (18%) |  |
| **Demographics** |  |  |  |  |  |  |
| Age years, median (IQR) | 56 (53; 58) | 55 (52; 58) | 56 (52; 58) | 57 (55; 58) | 56 (52; 58) | 0.007 |
| Race, N (Col. %)^c^ |  |  |  |  |  | <0.001 |
| Black women | 139 (21%) | 30 (32%) | 63 (22%) | 26 (17%) | 20 (17%) |  |
| Black men | 117 (18%) | 14 (15%) | 40 (14%) | 21 (13%) | 42 (36%) |  |
| White women | 201 (31%) | 26 (28%) | 105 (38%) | 54 (34%) | 16 (14%) |  |
| White men | 191 (29%) | 23 (25%) | 72 (26%) | 56 (36%) | 40 (34%) |  |
| Education years, median (IQR) | 16 (14; 18.0) | 16 (14; 18) | 16 (15; 18) | 16 (14; 17) | 14 (13; 16) | <0.001 |
| Study center, N (Col. %) |  |  |  |  |  | <0.001 |
| Birmigham, AL | 172 (27%) | 49 (53%) | 70 (25%) | 33 (21%) | 20 (17%) |  |
| Minneapolis, MI | 250 (39%) | 32 (34%) | 99 (35%) | 72 (46%) | 47 (40%) |  |
| Oakland, CA | 226 (35%) | 12 (13%) | 111 (40%) | 52 (33%) | 51 (43%) |  |
| **Substance use exposure** |  |  |  |  |  |  |
| Tobacco smoking, N (Col. %) |  |  |  |  |  | <0.001 |
| never smoker | 340 (52%) | 75 (81%) | 175 (62%) | 59 (38%) | 31 (26%) |  |
| past smoker | 226 (35%) | 14 (15%) | 82 (29%) | 81 (52%) | 49 (42%) |  |
| current smoker | 82 (13%) | 4 (4%) | 23 (8%) | 17 (11%) | 38 (32%) |  |
| Cumulative tobacco exposure among ever smokers/ tobacco pack-years^e^, median (IQR) | 0.0 (0.0; 5.8) | 0.0 (0.0; 0.0) | 0.0 (0.0; 1.5) | 1.1 (0.0; 10.3) | 4.9 (0.0; 17.8) | <0.001 |
| Alcohol use |  |  |  |  |  |  |
| Cumulative alcohol use  among ever drinkers/ drink-years^f^, median (IQR) | 10.3 (2.1; 27.9) | 0.4 (0.0; 4.8) | 8.0 (1.9; 20.9) | 17.1 (5.8; 33.9) | 31.2 (13.0; 63.5) | <0.001 |
| Cum. lifetime binge episodes^g^/ binge drinking days, median (IQR) | 24 (0; 312) | 0 (0; 0) | 0 (0; 96) | 120 (0; 420) | 390 (48; 1332) | <0.001 |
| Cannabis use status, N (Col. %)^d^ |  |  |  |  |  | <0.001 |
| never user | 93 (14%) | 93 (100%) | 0 (0%) | 0 (0%) | 0 (0%) |  |
| past user | 451 (70%) | 0 (0%) | 278 (99%) | 128 (82%) | 45 (38%) |  |
| current user | 104 (16%) | 0 (0%) | 2 (1%) | 29 (18%) | 73 (62%) |  |
| Cumulative cannabis exposure / cannabis years^a^, median (IQR) | 0.2 (0.0; 1.9) | 0.0 (0.0; 0.0) | 0.1 (0.0; 0.2) | 1.1 (0.8; 1.9) | 6.3 (3.6; 10.5) | <0.001 |
| Illicit drug use |  |  |  |  |  |  |
| Current use^h^   - Cocaine, crack, speed or methamphetamine, N (Col. %) | 22 (3%) | 1 (1%) | 2 (1%) | 5 (3%) | 14 (12%) | <0.001 |
| - Heroin, N (Col. %) | 0 (0%) | 0 (0%) | 0 (0%) | 0 (0%) | 0 (0%) |  |
| **Physical Activity**  Physical Activity Score^i^, median (IQR) | 287.5 (145.0; 504.0) | 223.0 (108.0; 390.0) | 287.0 (151.0; 491.5) | 325.0 (149.5; 545.5) | 265.0 (145.0; 519.2) | 0.067 |
| **Anthropomorphic variable**  BMI^j^, median (IQR) | 27.6 (24.5; 31.2) | 27.8 (25.0; 32.0) | 26.8 (24.2; 30.9) | 27.9 (24.5; 31.2) | 28.3 (25.2; 32.1) | 0.251 |
| **Psychological variable**  Depression, current CES-D >=16/30^k^, N (Col. %) | 71 (11%) | 9 (10%) | 24 (9%) | 21 (13%) | 17 (14%) | 0.129 |

BMI = body mass index; CARDIA = Coronary Artery Risk Development in Young Adults study; Col. % = column percentage, N = number of participants; +-SD = upper and lower limit of standard deviation

aSelf-reported cumulative exposure to cannabis joints in cannabis-years; 1 cannabis-year of exposure = 365 days of cannabis use (1 year × 365 days/y).

bP-values are from Kruskal-Wallis rank test for age, years of education, pack-years, drink-years, age started smoking, BMI and physical activity, from a χ2 test for race, study site,

current smoking status, CES-D, and cumulative binge drinking categories, and from a Fisher exact test for cannabis and illicit drug use categories.

cBy design, the CARDIA study sampled self-identified white men, white women, black men and black women in roughly equal numbers for participation in the study.

dCategories based on the answer to the question: “During the last 30 days, on how many days did you use marijuana?”

eSelf-reported cumulative exposure to cigarettes in pack-years: 1 pack-year of exposure = 7300 cigarettes (1 year × 365 days/y × 1 pack/d × 20 cigarettes/pack).

fCumulative alcohol use in drink-years: 1 drink-year is the total amount of ethanol consumed by a person who had 1 alcoholic drink per day for 1 year (1 drink-year = 17.24 ml of ethanol/drink x 1 drink/d x 365 days/y = 6292.6 ml of ethanol).

gBinge drinking days, defined as ≥5 drinks per day. If bingeing were to be constant over 30 years in one individual, 300 binge drinking days would correspond to 10 days of bingeing each year for 30 years.

hCurrent use, defined as any use within the last 30 days. Cocaine included all forms of cocaine, like crack, powder, free base; amphetamines included speed, uppers, and methamphetamines.

iPhysical activity, measured with the CARDIA Physical Activity History questionnaire, which queries the amount of time per week spent performing 13 categories of leisure, occupational, and household physical activities over the past 12 months.

jCalculated as weight in kilograms divided by height in meters squared.

kSelf-reported depression was measured every five years, starting at the Year 5 visit, on the Center for Epidemiologic Studies Depression scale (CES-D).18 A score of ≥16 was the cut-off for both sexes, indicating clinically significant depressive symptoms.

### **Appendix Table 2**: Distribution of hippocampal volume, fractional anisotropy (FA) and cerebral blood flow (CBF) at Year 30 and cumulative exposure to cannabis in ‘cannabis-years’ among 648 CARDIA participants at the Year 30 MRI sub-study, excluding participants with a history of stroke or TIA.

| **Variable** | **All** | **Never user** | **Ever Cannabis Use^a^** |  |  | **p-value^b^** |
| --- | --- | --- | --- | --- | --- | --- |
|  |  |  | 1 day to 0.5 cannabis years | 0.5 to < 2 cannabis years | > 2 cannabis years |  |
| Volume Hippocampus (mm^3^) | 3786.6 (3504.3; 4047.7) | 3717.5 (3468.8; 4055.1) | 3743.6 (3479.6; 4001.9) | 3829.0 (3572.1; 4105.8) | 3814.0 (3489.9; 4055.5) | 0.154 |
| FA Fornix | 0.265 (0.241; 0.289) | 0.271 (0.252; 0.290) | 0.261 (0.237; 0.285) | 0.268 (0.247; 0.293) | 0.262 (0.232; 0.282) | 0.011 |
| CBF Hippocampus (ml/100g/min) | 41.6 (36.2; 47.3) | 44.0 (39.2; 49.5) | 41.4 (36.2; 47.2) | 38.5 (34.8; 45.7) | 42.9 (36.5; 47.2) | 0.071 |

FA = fractional anisotropy, CBF = cerebral blood flow; CARDIA = Coronary Artery Risk Development in Young Adults study; N = number of participants; +-SD = upper and lower limit of standard deviation

aSelf-reported cumulative exposure to cannabis in cannabis-years; 1 cannabis-year of exposure = 365 days of cannabis use (1 year × 365 days/y).

bP-values from a Wald test. All P-values two sided.

### **Appendix Table 3:** Unadjusted and adjusted association between cumulative exposure to cannabis and hippocampal volume, fractional anisotropy (FA) and cerebral blood flow (CBF) at Year 30. 648 CARDIA participants at the Year 30 MRI sub-study excluding participants with a history of stroke or TIA.

| **Variable** | **Standardized difference in MRI measures (95% CI)^a^** | | |
| --- | --- | --- | --- |
|  | Unadjusted | Demographic adjusted | Fully adjusted |
| **Hippocampus volume** (mm^3^) |  |  |  |
| - Never user | Reference | Reference | Reference |
| - 1 day to 0.5 cannabis years | -62.25 (-134.20 to 9.69) | -95.63 (-169.06 to -22.19) | -81.65 (-157.69 to -5.61) |
| - 0.5 to < 2 cannabis years | -37.85 (-116.83 to 41.12) | -72.45 (-153.53 to 8.64) | -56.56 (-144.73 to 31.61) |
| - >2 cannabis years | -81.46 (-165.29 to 2.37) | -112.02 (-200.24 to -23.79) | -88.17 (-190.1 to 13.76) |
| P-value for trend^b^ | 0.22 | 0.049 | 0.18 |
| **Fornix FA** |  |  |  |
| - Never user | Reference | Reference | Reference |
| - 1 day to 0.5 cannabis years | -0.0080 (-0.0163 to 0.0003) | -0.0081 (-0.0163 to 0.0001) | -0.0059 (-0.0143 to 0.0025) |
| - 0.5 to < 2 cannabis years | 0.0003 (-0.0088 to 0.0094) | 0.0016 (-0.0074 to 0.0106) | 0.0035 (-0.0062 to 0.0133) |
| - >2 cannabis years | -0.0129 (-0.0225 to -0.0032) | -0.0093 (-0.0191 to 0.0005) | -0.0023 (-0.0136 to 0.0090) |
| P-value for trend^b^ | 0.005 | 0.009 | 0.055 |
| **Hippocampus CBF** (ml/100g/min) |  |  |  |
| - Never user | Reference | Reference | Reference |
| - 1 day to 0.5 cannabis years | -1.10 (-3.91 to 1.72) | 0.71 (-2.12 to 3.54) | - 1. (-1.48 to 4.38) |
| a0.5 to < 2 cannabis years | -3.45 (-6.65 to -0.26) | -1.12 (-4.33 to 2.10) | 0.02 (-3.46 to 3.50) |
| - >2 cannabis years | -0.99 (-4.32 to 2.33) | 1.97 (-1.46 to 5.39) | 2.46 (-1.52 to 6.43) |
| P-value for trend^b^ | 0.146 | 0.22 | 0.37 |

FA = fractional anisotropy, CBF = cerebral blood flow; CARDIA = Coronary Artery Risk Development in Young Adults study; N = number of participants; +-SD = upper and lower limit of standard deviation * Results from multivariable adjusted linear regression models. First unadjusted (only hippocampal volume adjusted for total intracerebral volume in unadjusted models), then adjusted for demographics (sex, race, age, education years, study center) and finally, for total physical activity score, current and cumulative alcohol use, cumulative cigarette smoking, self-reported lifetime illicit drug use (amphetamines, methamphetamines, cocaine, heroin), depression and BMI.

aLinear regression models determined the association between MRI scores and self-reported cumulative exposure to cannabis use. Negative standardized scores indicate smaller Volume, lower FA or lower CBF.

b P-values from a Wald test. All P-values two sided.

### **Appendix Table 4.** Association between cumulative exposure to cannabis and hippocampal volume, fractional anisotropy (FA) and cerebral blood flow (CBF) at Year 30, **stratified by sex, multivariable adjusted**. 648 CARDIA participants at the Year 30 MRI sub-study excluding participants with a history of stroke or TIA.

| **Variable** | **Standardized difference in MRI measures (95% CI)^a^** | |
| --- | --- | --- |
|  | Women | Men |
| **Hippocampus volume** (mm^3^) |  |  |
| - Never user | Reference | Reference |
| - 1 day to 0.5 cannabis years | -58.95 (-145.85 to 27.95) | -120.03 (-256.82 to 16.77) |
| - 0.5 to < 2 cannabis years | -42.52 (-147.35 to 62.30) | -89.29 (-242.94 to 64.37) |
| - >2 cannabis years | -37.10 (-164.90 to 90.70) | -156.01 (-324.16 to 12.14) |
| P-value for trend^a^ | 0.60 | 0.25 |
| **Fornix FA** |  |  |
| - Never user | Reference | Reference |
| - 1 day to 0.5 cannabis years | 0.0003 (-0.0102 to 0.0109) | -0.0110 (-0.0252 to 0.0032) |
| - 0.5 to < 2 cannabis years | 0.0090 (-0.0037 to 0.0217) | -0.0000 (-0.0160 to 0.0159) |
| - >2 cannabis years | 0.0011 (-0.0143 to 0.0165) | -0.0052 (-0.0226 to 0.0121) |
| P-value for trend^b^ | 0.30 | 0.18 |
| **Hippocampus CBF** (ml/100g/min) |  |  |
| - Never user | Reference | Reference |
| - 1 day to 0.5 cannabis years | 2.55 (-1.81 to 6.92) | -0.29 (-4.42 to 3.84) |
| - 0.5 to < 2 cannabis years | 0.46 (-4.81 to 5.74) | -1.63 (-6.50 to 3.24) |
| - >2 cannabis years | 3.89 (-2.56 to 10.34) | 0.66 (-4.55 to 5.86) |
| P-value for trend^b^ | 0.43 | 0.67 |

FA = fractional anisotropy, CBF = cerebral blood flow; CARDIA = Coronary Artery Risk Development in Young Adults study; N = number of participants; +-SD = upper and lower limit of standard deviation

* Main predictor (cumulative cannabis use) modeled flexibly.

a P-values from a Wald test. All P-values two sided.

### **Appendix Table 5:** Sensitivity analysis: Fully adjusted association between cumulative exposure to cannabis and **hippocampal** outcomes: **left and right hemisphere separatel**y, **adjusting for mirror star tracing test**, and **adjusting for additional CVRF**. 648 CARDIA participants with participation at the Year 30 MRI sub-study excluding participants with a history of stroke or TIA. **Stratified by ever tobacco smoking.**

| **NEVER TOBACCO SMOKER** | **Standardized difference in each MRI measures (95% CI)^a^** | | | | |
| --- | --- | --- | --- | --- | --- |
|  | Left Hippocampus | Right Hippocampus | Mirror star tracing test | CVRF^c^ |  |
| **Hippocampus volume** (mm^3^) |  |  |  |  |  |
| - Never user | Reference | Reference | Reference | Reference |  |
| - 1 day to 0.5 cannabis years | -74.16 (-168.86 to 20.55) | -87.70 (-185.33 to 9.94) | -79.78 (-174.30 to14.73) | -71.96 (-165.36 to 21.45) |  |
| - 0.5 to < 2 cannabis years | -36.61 (-156.72 to 83.51) | -43.00 (-166.84 to 80.83) | -31.12 (-151.11 to 88.87) | -29.66 (-147.80 to 88.49) |  |
| - >2 cannabis years | -8.40 (-178.08 to 161.29) | -34.59 (-209.52 to 140.35) | -19.39 (-187.41 to 148.62) | -20.28 (-187.71 to 147.15) |  |
| P-value for trend^b^/ R2 | 0.4/ 0.46 | 0.3/ 0.46 | 0.3/ 0.48 | 0.4/ 0.48 |  |
| **Fornix FA** |  |  |  |  |  |
| - Never user | Reference | Reference | Reference | Reference |  |
| - 1 day to 0.5 cannabis years | -0.00594 (-0.01551 to 0.00362) | -0.00431 (-0.01592 to 0.00730) | -0.00425 (-0.01454 to 0.00604) | -0.00522 (-0.01514 to 0.00470) |  |
| - 0.5 to < 2 cannabis years | 0.00209 (-0.01008 to 0.01425) | 0.00510 (-0.00967 to 0.01987) | 0.00477 (-0.00830 to 0.01784) | 0.00454 (-0.00808 to0.01716) |  |
| - >2 cannabis years | -0.01108 (-0.02814 to 0.00599) | 0.00272 (-0.01799 to 0.02344) | -0.00243 (-0.02062 to0.01577) | -0.00691 (-0.02474 to0.01093) |  |
| P-value for trend^b^/ R2 | 0.2/ 0.14 | 0.4/ 0.17 | 0.4/ 0.17 | 0.3/ 0.18 |  |
| **Hippocampus CBF** (ml/100g/min) |  |  |  |  |  |
| - Never user | Reference | Reference | Reference | Reference |  |
| - 1 day to 0.5 cannabis years | 0.30 (-3.72 to 4.32) | 0.88 (-2.92 to 4.67) | 0.71 (-3.15 to 4.58) | -0.30 (-4.12 to 3.53) |  |
| - 0.5 to < 2 cannabis years | -1.60 (-6.84 to 3.64) | -1.44 (-6.39 to 3.50) | -1.10 (-6.11 to 3.92) | -1.73 (-6.69 to 3.23) |  |
| - >2 cannabis years | -0.75 (-7.34 to 5.83) | -0.13 (-6.35 to 6.08) | -0.30 (-6.63 to 6.04) | -1.66 (-7.95 to 4.64) |  |
| P-value for trend^b^/ R2 | 0.9/ 0.14 | 0.6/ 0.15 | 0.7/ 0.17 | 0.9/ 0.19 |  |
| **EVER TOBACCO SMOKER** | **Standardized difference in each MRI measures (95% CI)^a^** | | | | |
|  | Left Hippocampus | Right Hippocampus | Mirror star tracing test | CVRF^c^ |  |
| **Hippocampus volume** (mm^3^) |  |  |  |  |  |
| - Never user | Reference | Reference | Reference | Reference |  |
| - 1 day to 0.5 cannabis years | -123.04 (-283.43 to 37.36) | -92.11 (-273.15 to 88.92) | -90.81 (-250.73 to 69.11) | -105.97 (-264.86 to 52.91) |  |
| - 0.5 to < 2 cannabis years | -150.09 (-316.79 to 16.61) | -65.84 (-253.99 to 122.32) | -87.74 (-254.90 to 79.42) | -95.01 (-260.77 to 70.75) |  |
| - >2 cannabis years | -165.50 (-340.19 to 9.19) | -105.68 (-302.85 to 91.49) | -99.91 (-275.45 to 75.64) | -146.41 (-320.39 to 27.58) |  |
| P-value for trend^b^/ R2 | 0.3/ 0.46 | 0.7/ 0.47 | 0.7/ 0.50 | 0.4/ 0.50 |  |
| **Fornix FA** |  |  |  |  |  |
| - Never user | Reference | Reference | Reference | Reference |  |
| - 1 day to 0.5 cannabis years | 0.00286 (-0.01428 to 0.02000) | 0.00282 (-0.01862 to 0.02425) | 0.00217 (-0.01598 to 0.02032) | 0.00254 (-0.01550 to 0.02059) |  |
| - 0.5 to < 2 cannabis years | 0.00880 (-0.00892 to 0.02652) | 0.01515 (-0.00701 to 0.03731) | 0.01076 (-0.00811 to 0.02963) | 0.01280 (-0.00593 to 0.03153) |  |
| - >2 cannabis years | 0.00379 (-0.01487 to 0.02244) | 0.01120 (-0.01214 to 0.03453) | 0.00808 (-0.01181 to 0.02797) | 0.00523 (-0.01454 to 0.02500) |  |
| P-value for trend^b^/ R2 | 0.6/ 0.19 | 0.2 / 0.20 | 0.4/ 0.20 | 0.2/ 0.21 |  |
| **Hippocampus CBF** (ml/100g/min) |  |  |  |  |  |
| - Never user | Reference | Reference | Reference | Reference |  |
| - 1 day to 0.5 cannabis years | 1.47 (-3.79 to 6.72) | 2.71 (-2.77 to 8.19) | 1.89 (-2.21 to 5.99) | 1.59 (-2.75 to 5.92) |  |
| - 0.5 to < 2 cannabis years | 1.18 (-4.29 to 6.64) | 2.13 (-3.57 to 7.83) | 0.51 (-3.55 to 4.56) | 0.77 (-3.69 to 5.23) |  |
| - >2 cannabis years | 4.73 (-1.24 to 10.69) | 6.00 (-0.22 to 12.22) | 4.27 (-0.33 to 8.88) | 3.92 (-0.94 to 8.78) |  |
| P-value for trend^b^/ R2 | 0.2/ 0.39 | 0.14/ 0.41 | 0.19/ 0.45 | 0.3/ 0.52 |  |

CARDIA = Coronary Artery Risk Development in Young Adults study;

* Results from multivariable adjusted mixed longitudinal models. Adjusted for demographics sex, race, age, education years, study center, current and cumulative alcohol use, cumulative cigarette smoking, self-reported lifetime illicit drug use (amphetamines, methamphetamines, cocaine, heroin), depression and BMI.

a Linear regression models determined the association between MRI scores and self-reported cumulative exposure to cannabis use. Negative standardized scores indicate smaller volume.

b P-values are from a Wald test.

c adjusted for blood pressure, blood cholesterol levels, fasting glucose level, Diabetes mellitus

### **Appendix Table 6:** Sensitivity analysis: Fully adjusted association between cumulative exposure to cannabis and hippocampal outcomes: **including participants with history of stroke or transient ischemic attack (TIA)**; only participants with **ongoing current** (in past 30 days) cannabis use; or **without current** cannabis use at their year 30 visit, separately. **Stratified by ever tobacco smoking.**

| **NEVER TOBACCO SMOKER** | **Standardized difference in MRI measures (95% CI)^a^** | | |
| --- | --- | --- | --- |
|  | Including participants with history of stroke or TIA  N = 346 | Current cannabis use  N = 34 | No current cannabis use  N = 306 |
| **Hippocampus volume** (mm^3^) |  |  |  |
| - Never user | Reference | n/a | Reference |
| - 1 day to 0.5 cannabis years | -72.51 (-163.24 to 18.23) | Reference | -85.18 (-178.89 to 8.53) |
| - 0.5 to < 2 cannabis years | -29.13 (-144.24 to 85.98) | -43.37 (-1387.27 to 1300.53) | -67.51 (-194.71 to 59.70) |
| - >2 cannabis years | -10.60 (-172.65 to 151.46) | -182.44 (-1429.41 to 1064.53) | 57.05 (-170.71 to 284.81) |
| P-value for trend^b^/ R2 | 0.4/ 0.48 | 0.9/ 0.69 | 0.2/ 0.49 |
| **Fornix FA** |  |  |  |
| - Never user | Reference | n/a | Reference |
| - 1 day to 0.5 cannabis years | -0.0051 (-0.0149 to 0.0047) | Reference | -0.0050 (-0.0150 to 0.0049) |
| - 0.5 to < 2 cannabis years | 0.0032 (-0.0094 to 0.0157) | 0.0312 (-0.0645 to 0.1269) | -0.0037 (-0.0173 to 0.0099) |
| - >2 cannabis years | -0.0032 (-0.0207 to 0.0143) | 0.0332 (-0.0597 to 0.1261) | -0.0032 (-0.0274 to 0.0210) |
| P-value for trend^b^/ R2 | 0.4/ 0.16 | 0.7/ 0.82 | 0.8/ 0.14 |
| **Hippocampus CBF** (ml/100g/min) |  |  |  |
| - Never user | Reference | n/a | Reference |
| - 1 day to 0.5 cannabis years | 0.50 (-3.25 to 4.25) | Reference | 1.10 (-2.76 to 4.95) |
| - 0.5 to < 2 cannabis years | -1.15 (-6.01 to 3.72) | -7.15 (-48.39 to 34.10) | -1.17 (-6.49 to 4.14) |
| - >2 cannabis years | -0.25 (-6.37 to 5.88) | 2.78 (-36.15 to 41.72) | -6.66 (-16.16 to 2.83) |
| P-value for trend^b^/ R2 | 0.9/ 0.16 | 0.5/ 0.83 | 0.3/ 0.15 |
| **EVER TOBACCO SMOKER** | **Standardized difference in MRI measures (95% CI)^a^** | | |
|  | Including participants with history of stroke or TIA  N = 314 | Current cannabis use  N = 70 | No current cannabis use  N = 238 |
| **Hippocampus volume** (mm^3^) |  |  |  |
| - Never user | Reference | n/a | Reference |
| - 1 day to 0.5 cannabis years | -92.09 (-253.08 to 68.90) | Reference | -95.47 (-266.61 to 75.68) |
| - 0.5 to < 2 cannabis years | -84.12 (-251.10 to 82.87) | 645.25 (57.39 to 1233.12) | -111.03 (-293.25 to 71.19) |
| - >2 cannabis years | -130.03 (-304.95 to 44.88) | 491.21 (-115.95 to 1098.38) | -132.76 (-341.74 to 76.21) |
| P-value for trend^b^/ R2 | 0.5/ 0.48 | 0.04/ 0.75 | 0.6/ 0.48 |
| **Fornix FA** |  |  |  |
| - Never user | Reference | n/a | Reference |
| - 1 day to 0.5 cannabis years | 0.0028 (-0.0153 to 0.0209) | Reference | 0.0057 (-0.0129 to 0.0244) |
| - 0.5 to < 2 cannabis years | 0.0126 (-0.0061 to 0.0314) | 0.0568 (-0.0174 to 0.1311) | 0.0125 (-0.0072 to 0.0323) |
| - >2 cannabis years | 0.0088 (-0.0109 to 0.0284) | 0.0491 (-0.0277 to 0.1259) | 0.0097 (-0.0129 to 0.0323) |
| P-value for trend^b^/ R2 | 0.2/ 0.21 | 0.3/ 0.54 | 0.5/ 0.23 |
| **Hippocampus CBF** (ml/100g/min) |  |  |  |
| - Never user | Reference | n/a | Reference |
| - 1 day to 0.5 cannabis years | 1.67 (-3.50 to 6.84) | Reference | 0.69 (-4.44 to 5.81) |
| - 0.5 to < 2 cannabis years | 1.31 (-4.08 to 6.70) | 17.65 (-2.61 to 37.90) | 0.30 (-5.31 to 5.91) |
| - >2 cannabis years | 4.50 (-1.33 to 10.32) | 24.92 (3.60 to 46.24) | 3.47 (-3.35 to 10.29) |
| P-value for trend^b^/ R2 | 0.2/ 0.41 | 0.03/ 0.90 | 0.7/ 0.44 |

CARDIA = Coronary Artery Risk Development in Young Adults study. n/a = nobody reported less than 0.5 cannabis years and current cannabis use at visit year 30.

* Results from multivariable adjusted mixed longitudinal models. Adjusted for demographics sex, race, age, education years, study center, current and cumulative alcohol use, cumulative cigarette smoking, self-reported lifetime illicit drug use (amphetamines, methamphetamines, cocaine, heroin), depression and BMI.

a Linear regression models determined the association between MRI scores and self-reported cumulative exposure to cannabis use. Negative standardized scores indicate smaller volume.

b P-values are from a Wald test.

### **Appendix Table 7:** Exploratory Analysis: **Distribution** of volume of the Amygdala and of the Prefrontal Cortex (PFC) at Year 30 and cumulative exposure to cannabis in ‘cannabis-years’^a^ among 648 CARDIA participants with participation in the Year 30 MRI sub-study excluding participants with a history of stroke or TIA. **Stratified by ever tobacco smoking.**

| **NEVER TOBACCO SMOKER** | **All** | **Never user** | **Ever Cannabis Use^a^** |  |  |
| --- | --- | --- | --- | --- | --- |
|  |  |  | 1 day to 0.5 cannabis years | 0.5 to < 2 cannabis years | > 2 cannabis years |
| N | 340 (100.0%) | 75 (22.1%) | 175 (51.5%) | 59 (17.4%) | 31 (9.1%) |
| Volume Amygdala (mm^3^), mean (SD) | 1,127 (130) | 1,112 (143) | 1,127 (128) | 1,130 (113) | 1,162 (135) |
| Volume PFC (mm^3^), mean (SD) | 24,090 (2,879) | 23,744 (3,002) | 24,084 (2,835) | 24,244 (2,708) | 24,660 (3,149) |
| **EVER TOBACCO SMOKER** | All | Never user | **Ever Cannabis Use^a^** | | |
|  |  |  | 1 day to 0.5 cannabis years | 0.5 to < 2 cannabis years | > 2 cannabis years |
| N | 308 (100.0%) | 18 (5.8%) | 105 (34.1%) | 98 (31.8%) | 87 (28.2%) |
| Volume Amygdala (mm3), mean (SD) | 1,102 (133) | 1,080 (121) | 1,091 (129) | 1,104 (136) | 1,118 (137) |
| Volume PFC (mm3), mean (SD) | 23,107 (3,027) | 22,379 (2,485) | 22,928 (2,965) | 23,171 (2,971) | 23,401 (3,266) |

CARDIA = Coronary Artery Risk Development in Young Adults study; N = number of participants; +-SD = standard deviation

aSelf-reported cumulative exposure to cannabis joints in cannabis-years; 1 cannabis-year of exposure = 365 days of cannabis use (1 year × 365 days/y).

bP-values are from 1-way analyses of variance. All P-values two sided.

### **Appendix Table 8:** Exploratory Analysis: Fully adjusted association between volume of the **Amygdala** or the **Prefrontal Cortex** (PFC) and **cumulative exposure to cannabis** at Year 30. 648 CARDIA participants with participation in the Year 30 MRI sub-study excluding participants with a history of stroke or TIA. Stratified by ever tobacco smoking. **Stratified by ever tobacco smoking.**

| **Variable** | **NEVER TOBACCO SMOKING** | **EVER TOBACCO SMOKING** |
| --- | --- | --- |
|  | **Standardized difference in each MRI measures (95% CI)^a^** | **Standardized difference in each MRI measures (95% CI)^a^** |
|  | Fully adjusted | Fully adjusted |
| **Amygdala volume** (mm^3^) |  |  |
| - Never user | Reference | Reference |
| - 1 day to 0.5 cannabis years | -6.39 (-32.50 to 19.71) | -29.08 (-84.29 to 26.13) |
| - 0.5 to < 2 cannabis years | -34.52 (-67.63 to -1.41) | -40.43 (-97.81 to 16.95) |
| - >2 cannabis years | -5.03 (-51.80 to 41.75) | -41.33 (-101.46 to 18.80) |
| P-value for trend^b^/ R2 | 0.15/ 0.57 | 0.5/ 0.47 |
| **PFC volume** (mm^3^) |  |  |
| - Never user | Reference | Reference |
| - 1 day to 0.5 cannabis years | 54.68 (-463.11 to 572.46) | -598.38 (-1620.80 to 424.04) |
| - 0.5 to < 2 cannabis years | -313.94 (-970.65 to 342.77) | -583.05 (-1645.67 to 479.57) |
| - >2 cannabis years | -188.38 (-1116.09 to 739.33) | -363.35 (-1476.88 to 750.19) |
| P-value for trend^b^/ R2 | 0.6/ 0.66 | 0.6/ 0.64 |

CARDIA = Coronary Artery Risk Development in Young Adults study;

* Results from multivariable adjusted mixed longitudinal models. Adjusted for demographics sex, race, age, education years, study center, current and cumulative alcohol use, cumulative cigarette smoking, self-reported lifetime illicit drug use (amphetamines, methamphetamines, cocaine, heroin), depression and BMI.

aLinear regression models determined the association between MRI scores and self-reported cumulative exposure to cannabis use. Negative standardized scores indicate smaller volume.

b P-values are from a Wald test.

### **Appendix Figure 1:** Association between cumulative cannabis use (modelled flexibly) and fractional anisotropy (FA) at Year 30. 648 CARDIA participants at the Year 30 MRI sub-study excluding participants with a history of stroke or TIA. **Left: never tobacco smoking, right: ever tobacco smoking.**


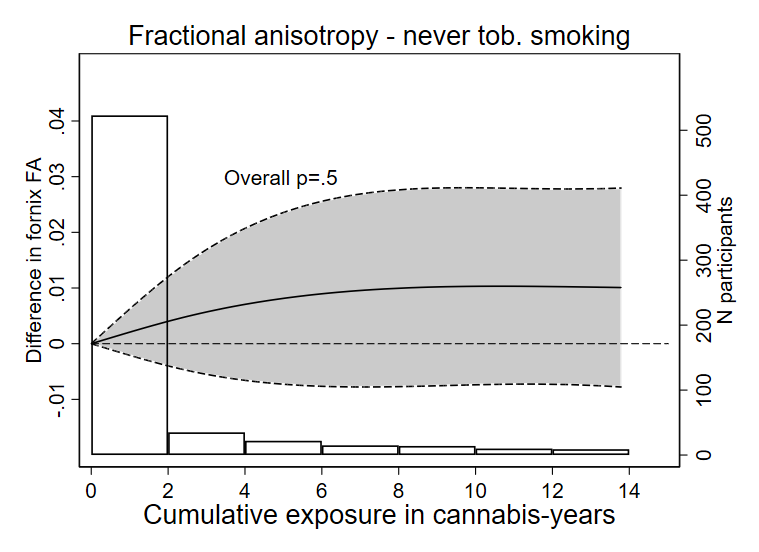

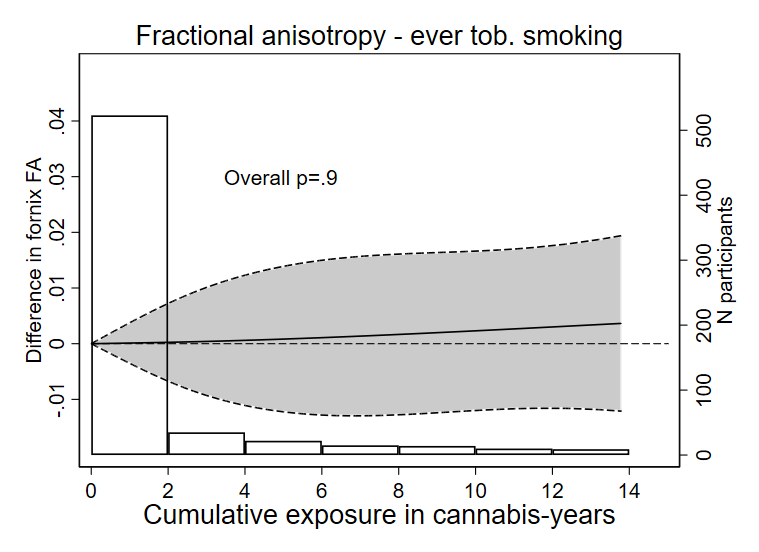


Results from multivariable adjusted linear regression models, using splines with three knots. Adjusted for demographics (sex, race, age, education years, study center), current and cumulative alcohol use, cumulative cigarette smoking, self-reported lifetime illicit drug use (amphetamines, methamphetamines, cocaine, heroin), depression and BMI. Cumulative exposure to cannabis expressed in cannabis-years, with 1 cannabis-year of exposure equivalent to 365 days of cannabis use.

**Appendix Figure 2**: Association between cumulative cannabis use (modelled flexibly) and cerebral blood flow (CBF) at Year 30. 648 CARDIA participants at the Year 30 MRI sub-study excluding participants with a history of stroke or TIA. **Left: never tobacco smoking, right: ever tobacco smoking.**
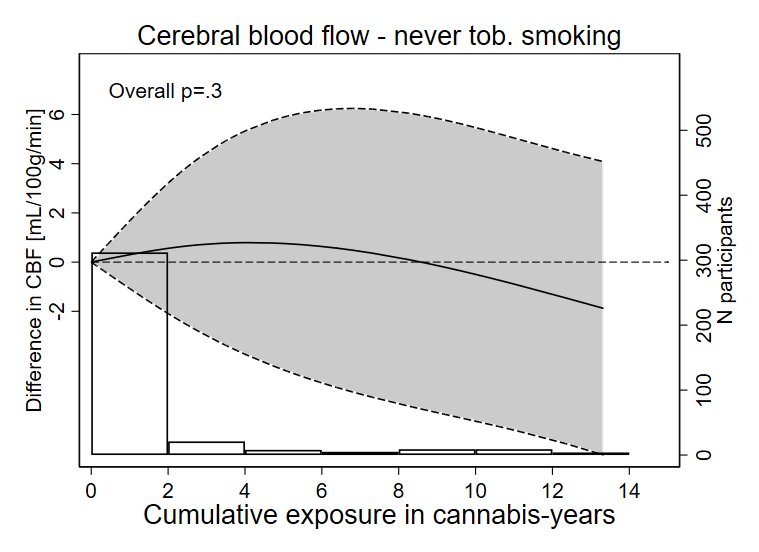

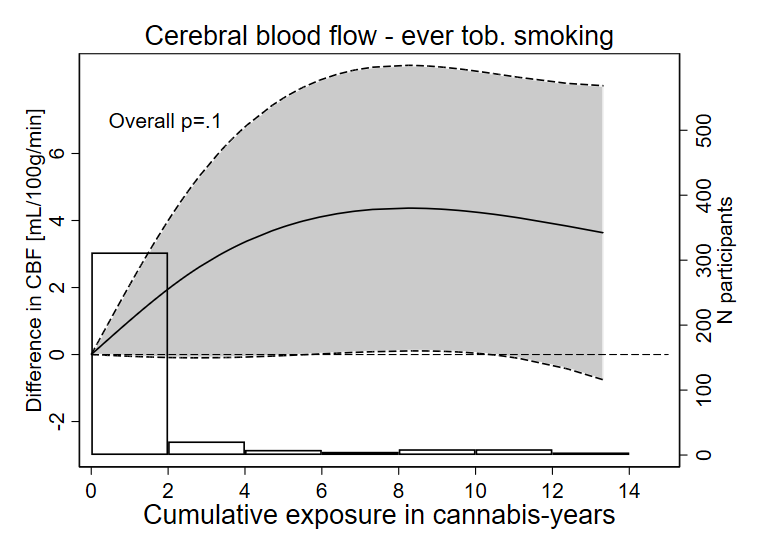


Results from multivariable adjusted linear regression models, using splines with three knots. Adjusted for demographics (sex, race, age, education years, study center), current and cumulative alcohol use, cumulative cigarette smoking, self-reported lifetime illicit drug use (amphetamines, methamphetamines, cocaine, heroin), depression and BMI. Cumulative exposure to cannabis expressed in cannabis-years, with 1 cannabis-year of exposure equivalent to 365 days of cannabis use.

# **eDiscussion**

**Cannabis dependency:** Another study found that cannabis users overall did not differ from controls in hippocampal volume or shape, but that cannabis-dependent users had significantly smaller right and left hippocampi relative to controls and non-dependent users[8]. Another recent study supports these findings. It found that the left hippocampal cornu ammonis subfield 1 (CA1) volumes were lower in dependent cannabis users compared with non-dependent cannabis users and with controls. Further, the left cornu ammonis subfield 3 (CA3) and left dentate gyrus volumes were lower in dependent versus non-dependent cannabis users (but not versus controls) [9]. This suggests dependency is associated with different brain anatomy, but cannabis use is not. This could explain our null findings as our study cannot distinguish between dependent and non-dependent users by study-design in CARDIA.

**Differences by sex:** In human brain imaging studies, certain brain markers have been found to differ between men and women due to cannabis use, indicating that there may be sex differences in susceptibility to the potential neurotoxic effects of cannabis [10, 11]. We found no significant association between cumulative cannabis use and hippocampal volume stratified by sex.

**Differences by hemisphere:** Some studies suggested a hippocampal volume reduction particularly in a single hemisphere, most commonly the left hippocampus [9, 12-14]. We did not find any association between cannabis use and hippocampal volume.

# **eReferences**

1. Pletcher, M.J., et al., *Association between marijuana exposure and pulmonary function over 20 years.* JAMA, 2012. **307**(2): p. 173-81.

2. Wagenknecht, L.E., et al., *Cigarette smoking behavior is strongly related to educational status: the CARDIA study.* Prev Med, 1990. **19**(2): p. 158-69.

3. Auer, R., et al., *Association Between Lifetime Marijuana Use and Cognitive Function in Middle Age: The Coronary Artery Risk Development in Young Adults (CARDIA) Study.* JAMA Intern Med, 2016. **176**(3): p. 352-61.

4. Alcoholism, N.I.o.A.A.a., *Helping Patients Who Drink Too Much: A Clinician's Guide.* 2005.

5. Kertesz, S.G., et al., *Illicit drug use in young adults and subsequent decline in general health: the Coronary Artery Risk Development in Young Adults (CARDIA) Study.* Drug Alcohol Depend, 2007. **88**(2-3): p. 224-33.

6. Leadbeater, B.J., M.E. Ames, and A.N. Linden-Carmichael, *Age-varying effects of cannabis use frequency and disorder on symptoms of psychosis, depression and anxiety in adolescents and adults.* Addiction, 2019. **114**(2): p. 278-293.

7. Launer, L.J., et al., *Vascular factors and multiple measures of early brain health: CARDIA brain MRI study.* PLoS One, 2015. **10**(3): p. e0122138.

8. Chye, Y., et al., *Alteration to hippocampal volume and shape confined to cannabis dependence: a multi-site study.* Addict Biol, 2019. **24**(4): p. 822-834.

9. Lorenzetti, V., et al., *Cannabis Dependence is Associated with Reduced Hippocampal Subregion Volumes Independently of Sex: Findings from an ENIGMA Addiction Working Group Multi-Country Study.* Cannabis Cannabinoid Res, 2024.

10. Francis, A.M., et al., *Interaction of sex and cannabis in adult in vivo brain imaging studies: A systematic review.* Brain Neurosci Adv, 2022. **6**: p. 23982128211073431.

11. Ketcherside, A., J. Baine, and F. Filbey, *Sex Effects of Marijuana on Brain Structure and Function.* Curr Addict Rep, 2016. **3**: p. 323-331.

12. Pagliaccio, D., et al., *Shared Predisposition in the Association Between Cannabis Use and Subcortical Brain Structure.* JAMA Psychiatry, 2015. **72**(10): p. 994-1001.

13. Yucel, M., et al., *Regional brain abnormalities associated with long-term heavy cannabis use.* Arch Gen Psychiatry, 2008. **65**(6): p. 694-701.

14. Paul, S. and S. Bhattacharyya, *Cannabis use-related working memory deficit mediated by lower left hippocampal volume.* Addict Biol, 2021. **26**(4): p. e12984.
